# PKC Delta Activation Promotes Endoplasmic Reticulum Stress (ERS) and NLR Family Pyrin Domain-Containing 3 (NLRP3) Inflammasome Activation Subsequent to Asynuclein-Induced Microglial Activation: Involvement of Thioredoxin-Interacting Protein (TXNIP)/Thioredoxin (Trx) Redoxisome Pathway

**DOI:** 10.3389/fnagi.2021.661505

**Published:** 2021-07-02

**Authors:** Manikandan Samidurai, Bharathi N. Palanisamy, Alejandra Bargues-Carot, Monica Hepker, Naveen Kondru, Sireesha Manne, Gary Zenitsky, Huajun Jin, Vellareddy Anantharam, Anumantha G. Kanthasamy, Arthi Kanthasamy

**Affiliations:** Department of Biomedical Sciences, Iowa Center for Advanced Neurotoxicology, Iowa State University, Ames, IA, United States

**Keywords:** ER stress, TXNIP, NLRP3, PKCδ, Parkinson’s disease

## Abstract

A classical hallmark of Parkinson’s disease (PD) pathogenesis is the accumulation of misfolded alpha-synuclein (αSyn) within Lewy bodies and Lewy neurites, although its role in microglial dysfunction and resultant dopaminergic (DAergic) neurotoxicity is still elusive. Previously, we identified that protein kinase C delta (PKCδ) is activated in post mortem PD brains and experimental Parkinsonism and that it participates in reactive microgliosis; however, the relationship between PKCδ activation, endoplasmic reticulum stress (ERS) and the reactive microglial activation state in the context of α-synucleinopathy is largely unknown. Herein, we show that oxidative stress, mitochondrial dysfunction, NLR family pyrin domain containing 3 (NLRP3) inflammasome activation, and PKCδ activation increased concomitantly with ERS markers, including the activating transcription factor 4 (ATF-4), serine/threonine-protein kinase/endoribonuclease inositol-requiring enzyme 1α (p-IRE1α), p-eukaryotic initiation factor 2 (eIF2α) as well as increased generation of neurotoxic cytokines, including IL-1β in aggregated αSyn_agg_-stimulated primary microglia. Importantly, in mouse primary microglia-treated with αSyn_agg_ we observed increased expression of Thioredoxin-interacting protein (TXNIP), an endogenous inhibitor of the thioredoxin (Trx) pathway, a major antioxidant protein system. Additionally, αSyn_agg_ promoted interaction between NLRP3 and TXNIP in these cells. *In vitro* knockdown of PKCδ using siRNA reduced ERS and led to reduced expression of TXNIP and the NLRP3 activation response in αSyn_agg_-stimulated mouse microglial cells (MMCs). Additionally, attenuation of mitochondrial reactive oxygen species (mitoROS) *via* mito-apocynin and amelioration of ERS *via* the eIF2α inhibitor salubrinal (SAL) reduced the induction of the ERS/TXNIP/NLRP3 signaling axis, suggesting that mitochondrial dysfunction and ERS may act in concert to promote the αSyn_agg_-induced microglial activation response. Likewise, knockdown of TXNIP by siRNA attenuated the αSyn_agg_-induced NLRP3 inflammasome activation response. Finally, unilateral injection of αSyn preformed fibrils (αSyn_PFF_) into the striatum of wild-type mice induced a significant increase in the expression of nigral p-PKCδ, ERS markers, and upregulation of the TXNIP/NLRP3 inflammasome signaling axis prior to delayed loss of TH^+^ neurons. Together, our results suggest that inhibition of ERS and its downstream signaling mediators TXNIP and NLRP3 might represent novel therapeutic avenues for ameliorating microglia-mediated neuroinflammation in PD and other synucleinopathies.

## Introduction

Parkinson’s disease (PD) is the second most common age-related neurodegenerative disorder (Mhyre et al., 2012). The classical clinical manifestations include motor symptoms such as resting tremors and muscular rigidity, as well as non-motor symptoms including constipation, sleep disturbances, and depression (Demaagd and Philip, 2015). While the precise etiology of PD is not well understood, exposure to environmental toxicants and genetic factors are implicated in disease development (Davie, 2008). PD originates from the progressive loss of nigral dopaminergic (DAergic) neurons, which is accompanied by the accumulation of misfolded aggregated αSyn (αSyn_agg_) within Lewy bodies and Lewy neurites which is a classical pathological hallmark of this disease (Dickson, 2012). Additionally, microglia-derived expression of pro-inflammatory cytokines such as IL-1β and TNF-α and the pro-oxidant NOS have been identified in the substantia nigra (SN), putamen, serum, and CSF of PD patients (Knott et al., 2000; Nagatsu et al., 2000; Frigerio et al., 2005; Teschke et al., 2014; Qin et al., 2016; Chen et al., 2018; Wijeyekoon et al., 2020). Numerous mechanisms have been implicated in PD pathogenesis, including mitochondrial dysfunction, endoplasmic reticulum stress (ERS), impaired proteostasis, and chronic neuroinflammation (Block et al., 2007) yet the exact mechanisms underlying increased vulnerability of nigral DAergic neurons to PD pathogenesis remains undefined. Interestingly, emerging evidence shows that the chronic microglia-driven inflammatory response plays a significant role in the progressive loss of nigral DAergic neurons and disease progression in animal models of PD (Gao et al., 2002; Qin et al., 2013; Duffy et al., 2018; Olanow et al., 2019). Therefore, it is imperative to better understand the contribution of chronic microglial activation to progressive nigral DAergic neurodegeneration in chronic neurodegenerative conditions including PD.

Microglia, the resident immune cells of the CNS, have been linked to the induction of the innate immune response in several neurodegenerative conditions including Alzheimer’s disease (AD; Webers et al., 2020; Leng and Edison, 2021). Depending on the local microenvironment, and in a brain region-specific manner, microglia carry out a number of functions in the CNS (Bachiller et al., 2018) including immune surveillance, neuronal apoptosis, synaptic plasticity, and pruning, as well as phagocytosis and repair (Russo and McGavern, 2015; Salter and Stevens, 2017; Leng and Edison, 2021). Following exposure to chemical insult or infection, DAMP (Damage-associated molecular pattern) receptors present on the surface can recognize abnormal protein aggregates, cellular debris, and pathogens, thereby mounting a microglial activation response (Heneka et al., 2015). Secretion of cytokines from persistently activated microglia in aged brains may promote a low-grade inflammation, thereby culminating in dysfunctional neuron-microglia cross talk (Norden and Godbout, 2013; Spittau, 2017). Additionally, these detrimental responses may amplify neuronal damage, thereby leading to an exaggerated disease pathology in the context of proteinopathies (Webers et al., 2020). Given that the microglial density is particularly high in the SN pars compacta (SNpc), it is plausible that microglia-mediated neuroinflammation may contribute to PD-associated αSyn pathology (Calabrese et al., 2018; Olanow et al., 2019).

Protein kinase C delta (PKCδ) is a redox-sensitive kinase involved in various processes related to proliferation, cell cycle progression, differentiation, and apoptosis (Gordon et al., 2016a). Previously, our lab demonstrated that PKCδ is highly expressed in the nigral DAergic neurons of PD patients (Zhang et al., 2007; Gordon et al., 2012) and that caspase-3-mediated proteolytic activation of PKCδ promotes neuronal apoptosis in preclinical experimental models of PD (Gordon et al., 2012). Emerging evidence from our lab and others supports a role for microglial PKCδ in the induction of reactive microgliosis in response to diverse inflammogens including αSyn_agg_ (Wen et al., 2011; Gordon et al., 2012, 2016a). Although PKCδ activation has been linked to ERS and associated cellular damage (Qin and Mochly-Rosen, 2008; Larroque-Cardoso et al., 2013), the exact relationship between PKCδ-mediated ERS and reactive microgliosis in the context of α-synucleinopathy remains elusive.

ER dysfunction, following the accumulation of misfolded proteins, triggers the unfolded protein response (UPR) that restores ER proteostasis within the protein secretory machinery (Hetz et al., 2020). The UPR pathway is mediated *via* three ER-localized parallel signaling pathways, namely: (1) serine/threonine-protein kinase/endoribonuclease inositol-requiring enzyme 1α (IRE1α), (2) protein kinase R (PKR)-like endoplasmic reticulum kinase (PERK)-eukaryotic translation initiation factor (EIFα), and (3) activating transcription factor 6 (ATF6), which contributes to the restoration of protein-folding defects *via* modifying the cellular transcriptional and translational machinery (Grootjans et al., 2016). On the other hand, prolonged ERS *via* maladaptive UPR signaling can cause inflammatory damage *via* NLRP3 inflammasome activation eventually leading to cellular demise including cell death (Mercado et al., 2013; Lebeaupin et al., 2018; Reverendo et al., 2019). In fact, the central pathological hallmark of PD, namely the accumulation of misfolded αSyn within Lewy bodies, has been linked to ER-associated DAergic neuropathology (Gitler et al., 2008; Bellucci et al., 2011; Colla et al., 2012). In recent years, the pivotal role of ERS in promoting the activation of the NLRP3 inflammasome has been demonstrated in various inflammation-related disease models (Chen X. et al., 2019; Ji et al., 2019). Numerous studies have demonstrated the beneficial effects of UPR modulation in attenuating PD-associated DAergic neuronal injury (Boyce et al., 2005; Colla, 2019). To the best of our knowledge, the exact role of the ERS-dependent mechanism in microglia-mediated neuroinflammation in the context of α-synucleinopathy remains a conundrum. Hence, in this study, we investigated the roles of PKCδ, ERS-dependent pro-inflammatory signaling events, and mitochondria-dependent ERS mechanisms in the microglial activation response in α-synucleinopathy models of PD. Our studies for the first time unveil a novel microglial ERS-driven inflammatory signaling pathway involving TXNIP and NLRP3 activation in the expression of the neurotoxic microglial activation state in the context of α-synucleinopathy and that inhibition of microglial ERS and the TXNIP/NLRP3 signaling axis may represent a novel and effective therapeutic strategy for PD treatment.

## Materials and Methods

### Chemicals and Biological Reagents

Modified Eagle’s medium (MEM), fetal bovine serum (FBS), L-glutamine, MitoSOX for reactive oxygen species (ROS) assay, IRDye-tagged secondary antibodies, Hoechst nuclear stain, penicillin, streptomycin, and other cell culture reagents were purchased from Invitrogen (Carlsbad, CA, USA). ATF-4, p-IRE1α, p-eIF2α, PKCδ, and p-PKCδ Y311 antibodies were purchased from Cell Signaling Technology (Danvers, MA, USA). A comprehensive list of the antibodies used in this study has been provided in Supplementary Table 1. NLRP3, ASC, and caspase-1 antibodies were purchased from AdipoGen (San Diego, CA, USA). The TrX and TXNIP antibody purchased from the Abcam (Cambridge MA). The β-actin antibody was purchased from Sigma-Aldrich (St. Louis, MO, USA). Mito-apocynin (MitoApo) was obtained from Dr. Kalyanaraman (Medical College of Wisconsin, Milwaukee, WI, USA). Protein A/G magnetic beads were purchased from Thermo Fisher Scientific (Waltham, MA). The CD11b magnetic separation kit was purchased from Stem Cell Technologies (Vancouver, Canada). The Duo-link proximity ligation assay reagents were purchased from Sigma Aldrich (St. Louis, MO). The Bradford protein assay was purchased from Bio-Rad Laboratories (Hercules, CA). The quick western kit was purchased from the LICOR (Lincoln, NE, USA).

### Human αSyn Purification and Aggregation

Ten milliliters of Luria broth medium with 100 μg/ml kanamycin was inoculated with BL21 (DE3) cells transformed with a pT7–7 plasmid encoding WT human αSyn from frozen stocks and incubated overnight at 37°C with shaking (pre-culture). The next day, the pre-culture was used to inoculate 1 liter of Luria broth/kanamycin medium. When the OD600 of the cultures reached 0.6, protein expression was induced with 1 mM isopropyl β-D-1-thiogalactopyranoside (Invitrogen), and the cells were further incubated at 37°C for 3 h before harvesting by centrifugation. Lysis was performed by resuspending the cell pellet in approximately 30 ml of 500 mM NaCl, 50 mM Tris HCl pH 7.6 using an Omni homogenizer at full speed for 5 min and ultra-sonicated with 30-s pulses followed by a 30-s pause, for a total ultra-sonication time of 3 min. Bacterial proteins were heat precipitated by incubating for 15 min in a 90°C water bath with gentle stirring. The solution was cooled by floating in ice water for 10 min then centrifuged at 10,000× *g* for 20 min. The supernatant was transferred to another tube and DNA was precipitated with streptomycin, 1 mg/ml of lysate, for 10 min at 4°C, and then ultracentrifuged at 24,500× *g*. The lysate was filtered through a 0.2-micron syringe filter into 3500 kD dialysis tubing and dialyzed against 20 mM Tris HCl pH 8.0 overnight. The lysate was collected from dialysis tubing and centrifuged at 15,000× *g* for 10 min, the supernatant was concentrated to a final volume of 10 ml, and the concentrate was filtered through a 0.2-μm syringe filter and applied to a GE Sephacryl S-200-HP column using the AKTA Pure FPLC unit. Fractions containing αSyn were identified by SDS-PAGE with Coomassie blue staining and pooled. Pooled fractions were then injected into a GE HiPrep Anion exchange column *via* AKTA Pure FPLC unit and eluted with a 20 mM Tris HCl and 2M NaCl solution. Fractions containing pure αSyn monomer were identified by SDS-PAGE and Coomassie blue staining was dialyzed against 20 mM Tris HCl pH 8.0 overnight. Protein concentration was determined *via* a NanoDrop 2,000 Spectrophotometer and aliquots of 1 mg were stored at −80°C until use.

### Endotoxin-Free Mouse αSyn Purification and Aggregation

Recombinant mouse αSyn proteins were purified as previously reported (Mao et al., 2016). The ToxinEraser endotoxin removal kit (GenScript) was used to remove the bacterial endotoxin following the manufacturer’s instructions. First, αSyn_agg_ was prepared in PBS to 5 mg/ml by agitating the monomers at 1,000 rpm at 37°C for 5 days continuously and then stored at −80°C until use. To obtain the preformed fibrils, the αSyn_agg_ stock is diluted from 5 mg/ml to 2 mg/ml in sterile PBS in a 1.5 ml Eppendorf tube. This preparation is sonicated using the Bioruptor Plus at high power for 10 cycles (30 s on, 30 s off) at a constant temperature of 10°C. The endotoxin levels post Toxin Eraser treatment were 0.08 EU/μg protein. All batches of αSyn were found to have endotoxin levels of <0.5 EU/μg protein.

### Transmission Electron Microscopy (TEM) Imaging

For TEM analysis αSyn_agg_ and αSyn_PFF_ were resuspended in 20 μl of sterile PBS and subsequently mixed with 2% uranyl acetate and incubated for 5 min at room temperature. Then, EM images were captured by adding 5 μl of the sample to carbon-coated copper grids and subsequently examined in a JEOL 2100 200-kV electron microscope operated at 80 kV. Additional analysis was performed using a Thermo Fisher Noran System 6 elemental analysis system.

### Cell Cultures and Culture Media

#### Mouse Microglial Cell Cultures

A wild-type (WT) mouse microglial cell (MMC) line (kind gift from Dr. D. Golenbock, University of Massachusetts) was derived by viral transduction of primary microglia. The maintenance media for the MMCs was DMEM-F12 containing 10% FBS and 1% penicillin, streptomycin, glutamine, and sodium pyruvate, while the experiments were conducted in the same media with reduced FBS (2%). Cell cultures were maintained at 37°C with 5% CO_2_ humidity.

#### MN9D DAergic Neuronal Cell Culture

The mouse DAergic MN9D cell line (RRID: CVCL_M067) was a kind gift from Dr. Syed Ali (National Center for Toxicological Research/Food and Drug Administration, Jefferson, AR) and cultured as described previously (Jin et al., 2011, 2014). Briefly, MN9D cells were grown in Dulbecco’s modified Eagle’s medium containing 10% fetal bovine serum (FBS), 2 mM L-glutamine, 1% penicillin, and 1% streptomycin and maintained at 37°C in a 5% CO_2_ atmosphere. Microglia conditioned media (MCM) was initially collected following exposure of MMCs to αSyn_agg_ with or without the addition of salubrinal (SAL). MN9D cells were subjected to the following MCM with the following treatments: (1) CON MCM, (2) αSyn_agg_ MCM, (3) SAL pretreatment followed by the αSyn_agg_ treatment MCM, and (4) SAL alone MCM. At the end of the treatment period, cells were subjected to MTS cell viability assay.

#### Primary Microglial Culture

Primary microglial (mouse PMG) cells were cultured from WT postnatal day 1 mouse pups based on a technique described by Sarkar et al. (2017) but with slight modification. Brains were harvested from the pups devoid of their meninges in DMEM/F12 supplemented with 10% heat-inactivated FBS, 1% penicillin, streptomycin, L-glutamine, nonessential amino acids, and sodium pyruvate. Brain tissues were then incubated in 0.25% trypsin-EDTA for 15 min with gentle agitation. Trypsinization was stopped by adding twice the volume of DMEM/F12 complete medium followed by triple-washing the tissues in complete media. Tissues were then triturated gently to prepare a single-cell suspension. To remove cell debris, the cell suspension was strained through a 70 μm nylon mesh cell strainer. The single-cell suspension was then seeded into T-75 flasks and incubated at 37°C with 5% CO_2_. As described by Sarkar et al. (2017), microglial cells were separated from mixed glial cultures using CD11b magnetic-bead separation to a 97% purity and were then allowed to recover for 48 h after plating. Upon isolation of microglia using the magnetic bead technique, the microglia-positive fraction was plated in T-75 flasks in growth media (DMEM/F12 complete medium). Following overnight incubation in growth media, the microglia were passaged again to begin various experiments.

### αSyn Internalization Assay

Approximately 25,000 primary microglial cells were plated onto PDL-coated coverslips. Primary mouse microglial cells were incubated with 1 μM of αSyn aggregates (rPeptide) for 1 h. At the end of the incubation period, cells were washed 3 times in 1× PBS and subsequently fixed with 4% PFA, washed in PBS, and cells were made permeable by incubating with 0.25% Triton X-100, and 0.05% Tween-20, blocked with 2% BSA for 1 h at RT. Cells were then immunostained with the respective primary antibodies IBA1 (1:1,000, Rabbit polyclonal) and human αSyn (1:500, mouse monoclonal) diluted in PBS containing 1% BSA and incubated overnight at 4°C followed by staining with Alexa Fluor 488 and Alexa Fluor 555 dye-conjugated secondary antibodies respectively. Nuclei were counterstained using Hoechst stain (10 μg/ml), and coverslips were mounted with Fluoromount medium and examined using an inverted fluorescence microscope (Nikon, Tokyo, Japan), and images were analyzed using Keyence BZ-X810.

### Animal Treatment

Six- to eight-week-old C57BL/6 mice were obtained from Charles River Labs (Wilmington, MA) and housed under standard conditions (22^o^C, 30% relative humidity, a 12-h light cycle, and *ad libitum* food and water). Given that males are at a higher risk of developing PD than women (Cerri et al., 2019), we utilized male mice in these studies. Mice were randomly divided into two groups (Control and αSyn_PFF_). An intrastriatal αSyn_PFF_-induced neuroinflammation model was used for this study as per Duffy et al. (2018). Briefly, the mouse was given continuous anesthesia throughout the procedure, and mouse-ear bars were placed firmly into each ear to secure the skull in place. The Angle 2 stereotaxic instrument was used with a 10-μl Hamilton syringe to inject the αSyn_PFF_ directly into the striatum (STR) at the following stereotaxic coordinates in relation to Bregma (mm): −2 ML, 0.5 AP, −4 DV. Prior to injection, αSyn_PFF_ is sonicated using a high-power bath sonicator system. The total volume of αSyn_PFF_ injected per site was 2 μl per mouse. The injection needle remained in place for an additional 2 min after completing the injection before it was gently withdrawn over a period of at least 30 s to minimize leakage. Mice were euthanized either 2 months after the initial intrastriatal injection for molecular experiments or after 6 months for TH analysis. The STR and SN were collected and stored at −80°C until further experimentation. The use of animals and all animal-related procedures in this study were approved and supervised by the Institutional Animal Care and Use Committee (IACUC) at Iowa State University, Ames, IA, USA.

### Immunohistochemistry: Diaminobenzidine Immunostaining

A mixture of 200 mg/kg ketamine and 20 mg/kg xylazine was used to deeply anesthetize mice for transcardial perfusion with freshly prepared 4% paraformaldehyde (PFA) solution. The perfused brains were immediately post-fixed in 4% PFA for 48 h as per our previous publication (Gordon et al., 2016b). Fixed brains were then cryoprotected in 10% sucrose before being sectioned into 30-μm coronal sections using a freezing microtome (Leica Microsystems, Wetzlar, Germany). DAB immunostaining in the coronal SN sections was performed as per Ghosh et al. (2013) with slight modification. Briefly, on the day of staining, 30-μm sections were washed with PBS, incubated with methanol containing 3% H_2_O_2_ for 30 min, washed with PBS 6x for 5 min each, and blocked with 10% normal goat serum, 0.5% Triton X-100 and 2% BSA in PBS for 1 h at room temperature (RT). Then, the sections were incubated with an anti-TH antibody (1:1,000, mouse monoclonal) overnight at 4°C and washed in PBS 6× for 10 min each. Biotinylated anti-rabbit secondary antibody was then used for 1 h at RT, washed in PBS 5× for 10 min each followed by incubation with avidin peroxidase and triple washed in PBS for 10 min each. Immunolabeling was observed using a DAB solution, which yielded a brown-colored stain. The stained sections were washed in PBS and carefully mounted on poly-L-lysine (PDL)-coated slides with organic solvent (DPX). Using the Stereo Investigator software (MBF Bioscience, Williston, VT, USA), the total numbers of TH^+^ neurons were counted stereologically using every sixth section of the SN (Ghosh et al., 2010). Samples were visualized using an Eclipse TE2000-U (Nikon, Tokyo, Japan) inverted fluorescence microscope, and images were captured using Keyence BZ-0023 (Keyence, Osaka, Japan).

### Immunohistochemistry and Immunofluorescence

Treated mice were sacrificed through transcardial perfusion performed using 4% PFA, and perfused brains were post-fixed in 4% PFA for 48 h. Next, 5-μm paraffin-embedded sections were cut on a microtome by the Department of Pathology, College of Veterinary Medicine, ISU. Sections underwent deparaffinization through a series of steps that includes xylene (x2), xylene:ethanol (1:1), 100% ethanol, 95% ethanol, 75% ethanol, ad 50% ethanol for 3 min each. Then, the sections underwent antigen retrieval using citrate buffer (10 mM sodium citrate, pH 8.5) for 30 min at 80°C. Next, the sections were blocked with 2% bovine serum albumin, 0.5% Triton X-100, and 0.05% Tween 20 in PBS for 1 h at RT. Sections were incubated with different primary antibodies such as anti-TXNIP (1:500; rabbit monoclonal), anti-eIF2α (1:500; rabbit monoclonal), and anti-Iba1 (1:500; goat polyclonal) overnight at 4°C. After washing with PBS, sections were incubated in appropriate secondary antibodies (Alexa Fluor 488 or 594 or 555) for 2 h followed by incubation with 10 μg/ml Hoechst 33342 for 5 min at RT to stain the nucleus. Then, the slides were mounted with Fluoromount medium (Sigma-Aldrich) on glass slides for visualization. Sections were viewed under a Keyence inverted fluorescence microscope.

Immunofluorescence studies in primary mouse microglia (PMG) cells were performed based on previously published protocols with few modifications (Ghosh et al., 2013). Approximately 25,000 cells were plated onto PDL-coated coverslips. After treatments, cells were fixed with 4% PFA, washed in PBS, and blocked with buffer (PBS containing 2% BSA, 0.5% Triton X-100, and 0.05% Tween-20) for 1 h at RT. Coverslips containing cells were probed with the respective primary antibodies IBA1 (1:1,000, goat polyclonal) and p-eIF2α (1:500, Rabbit polyclonal) diluted in PBS containing 1% BSA and incubated overnight at 4°C. Coverslipped cells were washed several times in PBS and incubated with Alexa Fluor 488 and Alexa Fluor 555 dye-conjugated secondary antibodies. Nuclei were counterstained using Hoechst stain (10 μg/ml), and coverslips were mounted with Fluoromount medium on glass slides for visualization. Samples were visualized using an inverted fluorescence microscope (Nikon, Tokyo, Japan), and images were captured using Keyence BZ-X810.

### Intracellular MitoSOX Assay

As a measure of oxidative stress, mitochondrial superoxide levels were estimated using MitoSox. Primary microglial cells (~20,000 cells per well) were seeded in a 96-well plate and treated with αSyn_agg_ at various time points after cell attachment. Following treatment, the cells were exposed to 5 μM MitoSOX dye and incubated for 20 min at 37°C, protected from light. The cells were then washed twice with Hank’s Buffered Salt Solution (HBSS) before measuring the fluorescence intensity using a Spectramax™ microplate reader at 510 and 580 nm of excitation and emission wavelengths, respectively.

### Mitochondrial Membrane Potential (MMP) Measurement

Mitochondrial function, a key indicator of cell health, can be assessed by monitoring changes in MMP. Membrane potential changes in the mitochondria were monitored by 5,5′,6,6′-tetrachloro-1,1′,3,3′-tetraethyl-benzimidazolcarbocyanineiodide (JC-1) staining as previously described (Ghosh et al., 2010). Briefly, the cells were plated at 20,000 cells/well into a 96-well black-walled, clear-bottom plate. The assay plates were incubated overnight at 37°C for cell adhesion. After the respective incubation times, JC-1 dye solution was added to each well, and the cells were incubated at 37°C, 5% CO_2_ for 30 min. The monomeric form of JC-1 dye in the cytosol was detected fluorometrically through the emission of green fluorescence at the excitation/emission wavelengths 485/535 nm and aggregates in healthy mitochondria were detected through the red fluorescence emitted at the excitation/emission wavelengths of 550/600 nm using a Spectramax™ microplate reader. The changes in MMP were calculated as the red/green ratio and expressed as a percentage of control.

### Microglial Nitric Oxide Detection

Nitric oxide production by primary microglial cells was measured by Griess assay (Sigma-Aldrich) as described previously. The cells were plated at 20,000 cells/well into a 96-well plate. The assay plates were incubated overnight at 37°C for cell adhesion and exposed to αSyn_agg_ at various time points (6, 12, 18, 24 h). At the end of each treatment, 100 μl of supernatant was added to an equal volume of Griess reagent per well of a 96-well plate. The samples were then incubated at 37°C at RT for 15 min until their color stabilized. The absorbance was measured at 540 nm using a Synergy 2 multimode microplate reader (BioTek Instruments, Winooski, VT, USA). Sodium nitrate was used as the standard to determine the nitrite concentration in the samples.

### MTS Assay

MTS assay using the Cell Titer 96^®^ Aqueous One Solution Cell Proliferation (MTS) assay kit was conducted based on the method described in our previous publications (Charli et al., 2016; Singh et al., 2018). Briefly, 20,000 cells/well were seeded in a 96-well tissue culture plate and exposed to 100 μl of treatment media, and the respective conditioned media was collected from the microglia. Cells were then incubated with the MTS dye for 45 min in a humidified, 5% CO_2_ atmosphere at 37°C. The supernatant was removed, the resulting formazan crystals were dissolved with 25 μl of dimethyl sulfoxide (Sigma-Aldrich), and the color change was measured spectrophotometrically at 490 nm. The values were expressed as % of control.

### Real-Time Quantitative Reverse Transcription PCR (qRT-PCR)

SYBR Green qRT-PCR was performed on the harvested cells and tissues as described by Samidurai et al. (2020). RNA was extracted using the TRIZOL reagent (Invitrogen, Carlsbad, CA, USA) as per the manufacturer’s protocol. The isolated RNA was converted into cDNA using a High-Capacity cDNA synthesis kit (Applied Biosystems, Waltham, MA, USA). The qRT-PCR was carried out on an Applied Biosystems QuantStudio 3 system with the SYBR master mix (Invitrogen, Carlsbad, CA, USA). QuantiTect Primer Assays (Qiagen, Germantown, MD, USA) for genes specific to the pro-inflammatory cytokines TNF-α, IL-1β (#330001), and IL-6 were used. Normalization was done with the housekeeping gene 18S rRNA. TNF-α and IL-6 were obtained from the Iowa State University DNA Facility ISU DNA Facility (sequences are provided in Supplementary Table 2), IL-1β (QT01048355), and 18S (#PPM57735E) purchased from the Qiagen. Dissociation curves were run to ensure a single amplicon peak in all the SYBR Green reactions. The results were graphed as fold change in the respective gene expression calculated using the ΔΔCt method.

### Western Blotting (WB)

Cells and tissue were lysed and homogenized using modified RIPA buffer and subjected to fluorescent immunoblotting as previously described (Gordon et al., 2016b; Samidurai et al., 2020). Normalized protein (30 μg) from each sample was loaded into their respective wells and separated using a 12% SDS-PAGE gel. The proteins were then transferred to a nitrocellulose membrane, and the nonspecific-binding sites were blocked for 1 h at RT using an Odyssey blocking buffer (LI-COR, Lincoln, NE, USA). Membranes were then incubated overnight at 4°C in one of the following primary antibodies p-PKCδ, PKCδ, ATF-4, p-IRE1α, p-eIF2α (1:1,000, Rabbit monoclonal), NLRP3 (1:1,000, mouse monoclonal), ASC (1:1,000, rabbit polyclonal) caspase-1 (1:1,000, mouse polyclonal), TXNIP and TrX (1:1,000, Rabbit polyclonal). Next, the membranes were triple-washed with PBS, and then the membranes were visualized using IRDye-tagged secondary antibodies (incubated for 1 h at RT) on an LI-COR Odyssey infrared imaging system. To avoid variation in loading, the same blots were stripped *via* incubation in NewBlot Stripping Buffer for 30 min in RT and incubated with an anti-actin antibody; β-actin (1:10,000, mouse monoclonal) was used as a loading control to confirm the loading of equal protein concentration in each lane. Full blot images have been provided in the Supplementary Figure 4.

### Co-immunoprecipitation (Co-IP) Studies

Immunoprecipitation studies were carried out as described previously but with minor modifications (Filhoulaud et al., 2019; Panicker et al., 2019). Approximately 6 × 10^6^ MMCs were seeded and treated with αSyn_agg_ or vehicle for 18 h. Subsequently, for TXNIP immunoprecipitation experiments, cells were collected and spun down at 5,000× *g* and the cell pellets were resuspended and lysed with TNE buffer (10 mM Tris-HCl, pH 7.5, 1% Nonidet P-40, 0.15 M NaCl, 1 mM EDTA, and 1:1,00 protease inhibitor mixture), and kept on ice for 30 min. The lysates were then centrifuged at 17,400× *g* for 35 min at 4°C to remove the debris. The supernatant protein concentration was measured and normalized using the Bradford method. For the input fraction, approximately 50 μg protein was used. Approximately for immunoprecipitation analysis, 500 μg protein per sample in 500 μl TNE buffer was used. The extracted proteins were incubated with 5 μg of anti-TxNIP antibody and placed on an orbital shaker at 4°C overnight. The following day, bound proteins were recovered following the addition of protein A/G magnetic beads (Thermo-fisher) and subsequent placement on an orbital shaker for 1 h at RT. Protein A/G magnetic beads were centrifuged and subsequently subjected to four washes for 2–3 min each with TNE buffer. The bound proteins were eluted from the beads by boiling in Laemmli buffer for 10 min at 97°C. The eluted proteins were collected by centrifuging at 5,000 rpm for 5 min and separated on 12% SDS-PAGE gels and followed by immunoblotting using antibodies against TXNIP. Quick Western detection was used to avoid the visualization of denatured IgG. Membrane images were captured on an LI-COR Odyssey imaging system.

### Duo-link Proximal Ligation Assay (PLA)

Proximity ligation assay (PLA) was carried out in PMG according to the manufacturer’s protocol. Briefly, 10,000 primary microglial cells were seeded on PDL-coated coverslips in 96 well culture plates. After treatment of PMG with αSyn for 18 h, cells were washed with PBS and fixed using 4% PFA, blocked with blocking buffer (PBS containing 1.5% BSA, 0.5% Triton X-100, and 0.05% Tween-20), and subsequently incubated with antibodies against NLRP3 and TXNIP at 4°C overnight. Secondary antibodies conjugated with positive and negative oligonucleotides (PLA probes) were then incubated with the cells. Next, ligase was added and these oligonucleotides hybridize to the PLA probes, and when close enough, form a closed circle. In the DNA circle, one of the antibody-conjugated DNA probes serves as a primer for rolling-circle amplification (RCA), and a repeated sequence (concatemeric) product was generated when DNA polymerase and nucleotides were added. Fluorescently labeled oligonucleotides proceed to bind to the RCA product, allowing visualization of the protein-protein interaction as single dots *via* fluorescence microscopy (Wang et al., 2015). The protein-protein interactions were visualized as green puncta and analyzed by fluorescence microscopy using a Keyence microscope (with 60X objective). The PLA signals were further quantified using the ImageJ software according to the previous publication (Gomes et al., 2016).

### siRNA Transfection

The PKCδ siRNA was purchased from Santa Cruz Biotech (#SC-36246; Dallas, TX, USA), and TXNIP siRNA was purchased from life technologies (Ambion #4390771; Carlsbad, CA, USA). Lipofectamine 3,000 reagent was used for all the siRNA transfections according to the manufacturer’s protocol. One-day-old MMCs seeded at the density of 1.5 × 10^6^ in 6-well plates were used for transfection studies. Next, 700 pM of PKCδ siRNA, or 1.5 nM of TXNIP siRNA, or an equal amount of scrambled siRNA mixed with 5 μl of Lipofectamine 3,000 was added to the respective wells. The cells were then incubated with siRNA for 48 h, after which cells were treated with αSyn_agg_ for 24 h. Immunoblots were performed to check for the efficiency of target gene knockdown as well as for the determination of inflammation and ERS markers.

### Data Analysis

Data were analyzed using a two-tailed *t*-test (two groups) or one-way or two-way ANOVA (multiple groups) followed by Bonferroni’s *post hoc* analysis (PRISM 6.0 software, GraphPad, La Jolla, CA, USA), and are represented as mean ± SEM. Differences were considered statistically significant for p-values ≤ 0.05.

## Results

### Aggregated αSyn (αSyn_agg_) Enhanced Mitochondrial Impairment, PKCδ Activation, and ER Stress (ERS) *In vitro* in Mouse Primary Microglial Cells

**Figure 1:**
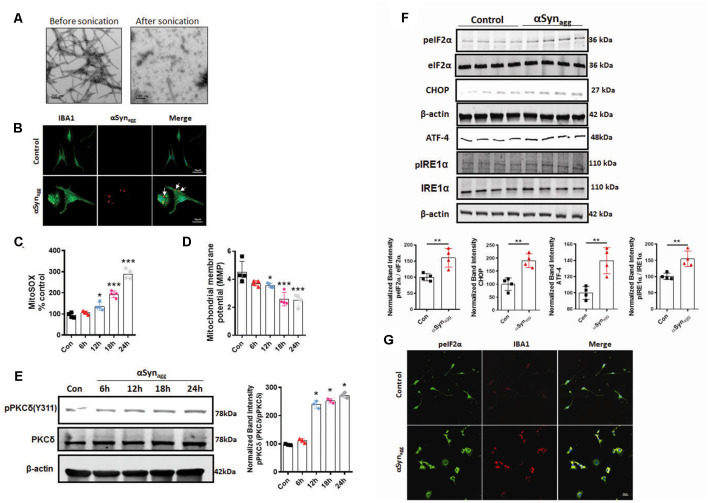
Time-dependent mitochondrial dysfunction, PKCδ activation, and endoplasmic reticulum stress (ERS) markers in αSyn_agg_-stimulated mouse primary microglia. Mouse primary microglial cells were exposed to 1 μM of αSyn_agg_ for increasing durations (6, 12, 18, and 24 h) and mitochondrial superoxide generation was measured using MitoSOX dye-based spectrofluorometric analysis. **(A)** Generation of human preformed fibrils (PFF) αSyn and validation of uptake of αSyn_agg_ by primary microglia. Representative scanning transmission electron microscopy (STEM) images of αSyn_PFF_ before scale bar = 200 (nm) and after scale bar = 200 (nm) sonication. Even after sonication the αSyn_PFF_ still retain their beta-sheet conformation. **(B)** Internalization of αSyn_agg_ in primary microglia. Representative immunohistochemical images of mouse primary microglia depicting uptake of hu-αSyn as determined using antibodies against human αSyn (red) and microglial marker-IBA-1 (green) in primary microglia treated with αSyn_agg_ for 1 h. Internalized human αSyn, visualized as red intracellular puncta), Scale bars, 15 μm. **(C)** MitoROS generation increased in a time-dependent manner in αSyn_agg_-treated mouse primary microglia cells. Data shown are the mean ± SEM from at least three independent experiments. **(D)** Assessment of mitochondrial membrane potential [MMP (ΔΨm)] using JC-1 spectrophotometric plate reader analysis. MMP decreased time-dependently post mouse PMG stimulation with αSyn_agg_. Data shown are the mean ± SEM from at least three independent experiments. **(E)** Representative immunoblots and densitometric evaluation of p-PKCδ (Y311) in the whole-cell lysates indicating a time-dependent increase in PKCδ phosphorylation in αSyn_agg_-treated mouse PMG. Data shown are the mean ± SEM from at least three independent experiments. **(F)** Representative immunoblots and densitometric analyses of mouse PMG cells treated with or without αSyn_agg_ for 24 h revealing significantly increased ATF-4, p-IRE1α, and and CHOP expression levels. The immunoblot is representative of at least three independent experiments. **(G)** Representative images from dual immunohistochemical staining for p-eIF2α (Red) IBA1 (green) in primary microglia treated with αSyn_agg_ for 24 h. Nuclei were counterstained with Hoechst stain (Blue). Results represent three independent experiments. Scale bar = 20 μm. Data were analyzed using one-way ANOVA followed by Bonferroni’s *post hoc* analysis and two-tailed *t*-test. Asterisks (***p < 0.001, **p < 0.01 and *p ≤ 0.05) indicate significant differences between control and treatment groups.

Lewy bodies, which represent a classical pathological hallmark of PD and DLB exhibits microglial and neuronal inclusions enriched with αSyn_agg_ (Fellner et al., 2011; Hoffmann et al., 2019). Given that αSyn_agg_ positive inclusions appear prior to the development of clinical signs (Braak et al., 2006) and that they are taken up by the microglia and contribute to the templated conversion of endogenous monomeric αSyn (Earls et al., 2019; Pike et al., 2021). We initially characterized the αSyn fibrils. Therefore, in this study, we used the human αSyn_agg_ which was found to induce microglial activation response consistent with a previous finding from our lab (Panicker et al., 2019). In our previous studies, we examined αSyn fibrils using the thioflavin T assay to determine the extent of fibrillar content in the αSyn_PFF_ fractions. Consistent with a previous report our studies revealed that a majority of the αSyn_agg_ were composed of fibrillar species (Manne et al., 2019). We further validated the confirmation of fibrillar αSyn species using transmission electron microscopy (TEM; Figure 1A) prior to microglia treatment studies. Moreover, we found internalization and microglial activation capabilities by human αSyn_agg_ in mouse primary microglia as previously reported (Panicker et al., 2019). In fact, immunofluorescence staining verified that treatment of primary microglia with 1 μM αSyn_agg_ led to its internalization (Figure 1B) as determined using an antibody against human total αSyn, consistent with a previous finding from our lab (Panicker et al., 2019). Given that αSyn aggregation reportedly induces mitochondrial dysfunction, thereby contributing to disease pathology (Smith et al., 2005) next, we investigated the temporal pattern of mitoROS generation, mitochondrial membrane potential (MMP) collapse, and PKCδ activation in mouse primary microglia (mouse primary microglia) stimulated with 1 μM αSyn_agg_ based on previous reports (Boza-Serrano et al., 2014; Lee et al., 2018; Panicker et al., 2019) for increasing durations (6, 12, 18, 24 h; Hoffmann et al., 2016; Sarkar et al., 2020). Following treatment of primary microglial cells with 1 μM of αSyn_agg_ robust mitochondrial superoxide generation, mitochondrial membrane potential (MMP) generation and nitrite release were evidenced as determined by MitoSOX, JC-1, and Griess spectrophotometric plate reader assays, respectively. The cells treated with αSyn_agg_ exhibited a significant (*p* < 0.001) time-dependent increase in mitochondrial (mito)ROS generation with an accompanying dissipation of MMP (Figures 1C,D), as well as nitrite release in a time-dependent manner (Supplementary Figure 1B) as compared to vehicle-treated cells. These results indicate that αSyn_agg_ induced mitochondrial dysfunction in primary microglia *via* a mitochondrial oxidative stress mechanism and accompanying collapse of MMP.

Previous reports show that PKCδ is a critical regulator of the proinflammatory microglial activation state in response to diverse inflammogens including αSyn, TNF-α, and LPS (Wen et al., 2011; Gordon et al., 2016a). Next, we examined whether activated PKCδ, a redox-sensitive kinase, is associated with mitochondrial dysfunction. Our WB studies revealed that αSyn_agg_ stimulation of mouse primary microglia resulted in a pronounced time-dependent activation of PKCδ as evidenced by prominent PKCδ phosphorylation at site Tyr-311 at 12 h which remained elevated for the remainder of the treatment duration as compared to vehicle-treated cells (Figure 1E), suggesting that mitochondrial oxidative stress may serve as a trigger for PKCδ activation consistent with previous reports (Majumder et al., 2001; Steinberg, 2015). These data collectively suggest that PKCδ activation contributes to microglia activation presumably *via* mitochondrial oxidative stress mechanism. Several reports link misfolded αSyn-induced PD pathology and ERS. For example, the overexpression of A53T αSyn promotes ER dysfunction, thereby eliciting pronounced ER stress (Smith et al., 2005; Karim et al., 2020), and blockage of ERS was found to protect against A53T αSyn-induced cell death (Boyce et al., 2005). Moreover, aberrant generation of mitoROS has been shown to contribute to ERS (Liu et al., 2019). Therefore, we further examined the magnitude of expression of ERS markers in αSyn_agg_ stimulated mouse primary microglia. As anticipated, the upregulation of ERS markers, including p-IRE1α, p-eIF2α, CHOP, and ATF-4, were observed in αSyn_agg_-treated mouse primary microglia as compared with vehicle-treated cells (Figure 1F). Likewise, immunofluorescence analysis showed that αSyn_agg_ increased the p-eIF2α immunoreactivity in the primary microglia cells as compared to the vehicle treated cells further supporting the central role of ERS in microglial activation response in response to αSyn_agg_ (Figure 1G). To ensure that the observed effects were not due to cellular toxicity, we performed a cell viability assay using an MTS assay. MTS revealed little or no evidence of cell death at a concentration of 1 μM αSyn_agg_ (Supplementary Figure 1A). Given that αSyn concentrations ranging between 0.5–5 μM have been widely used in various cell culture studies and that the synaptic concentration of αSyn is believed to fall within the range of 2–4 μM (Konno et al., 2012; Westphal and Chandra, 2013; Boza-Serrano et al., 2014; Domert et al., 2016; Lee et al., 2018; Hoffmann et al., 2019), we used 1 μM αSyn_agg_ in the remainder of the experiments unless otherwise stated. These results indicate that our αSyn_agg_ proteins are capable of inducing a reactive microglial activation state *via* mitochondrial oxidative stress and PKCδ activation mechanism as well as ERS in mouse primary microglia.

### αSyn_agg_ Increased the Expression of TXNIP and NLRP3 Inflammasome Components With an Accompanying Elevation of Pro-inflammatory Cytokine Generation in Mouse Primary Microglia

**Figure 2:**
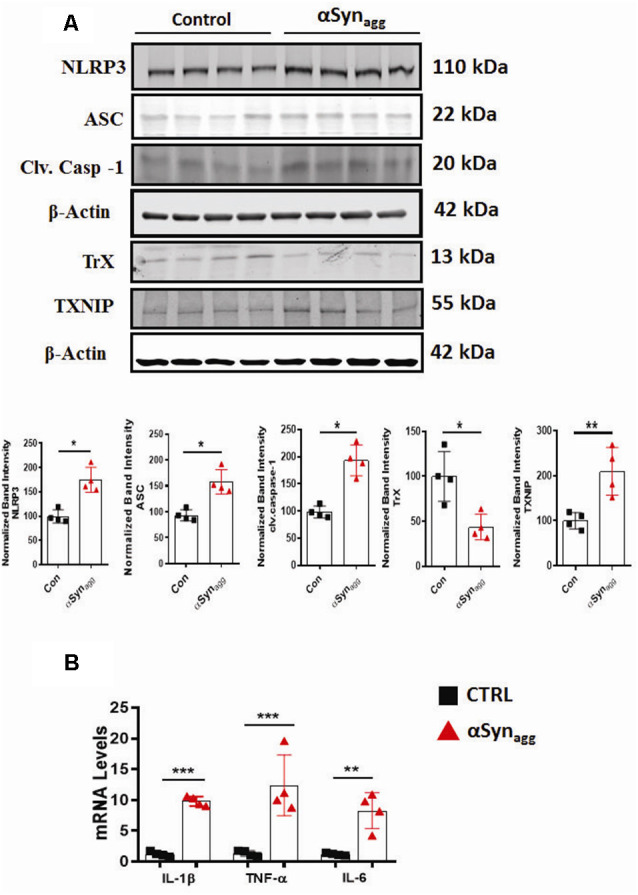
Activation of TXNIP and TrX downregulation positively correlates with the induction of NLRP3 inflammasome activation markers and pro-inflammatory cytokine generation in αSyn_agg_-stimulated mouse PMG. **(A)** Western blot analysis showing increased expression of NLRP3, ASC, caspase-1, TXNIP and downregulation of TrX expression following stimulation of mouse primary microglia with αSyn_agg_ for 24 h and quantification of the expression levels of the aforementioned markers. β-actin was used as an internal loading control. Data presented as mean ± SEM from four independent experiments. **(B)** qRT-PCR analysis showing increased gene expression of pro-inflammatory cytokines including TNF-α, IL-1β, and IL-6 after αSyn_agg_ stimulation for the indicated treatment period. Data are presented as the mean ± SEM and representative of three independent experiments. Data were analyzed using two-tailed *t*-test. Asterisks (***p < 0.001, **p < 0.01, and *p ≤ 0.05) indicate significant differences between control and treatment groups.

Thioredoxin-interacting protein (TXNIP), also known as thioredoxin-binding protein-2 or vitamin D3-upregulated protein 1, has been shown to be an endogenous antagonist of the antioxidant protein thioredoxin (Trx) (Zhao et al., 2017b). In oxidatively stressed cells, in response to ERS, TXNIP has been linked to NLRP3 inflammasome activation (Wang C. Y. et al., 2019). To test whether TXNIP/Trx expression levels are altered by αSyn_agg_, we treated primary microglia with αSyn_agg_. As predicted, TXNIP expression was significantly increased while Trx expression levels were downregulated in mouse primary microglia stimulated with αSyn_agg_ (Figure 2A) as compared to controls. We next examined whether TXNIP upregulation is associated with increased expression of NLRP3 inflammasome activation markers as well as inflammatory cytokine generation. Our studies indicate that αSyn_agg_ treatment increased protein expression of NLRP3 inflammasome components alongside expression of the pro-inflammatory cytokines IL-1β, IL-6, and TNF-α in αSyn_agg_-treated mouse primary microglia as compared to vehicle (con) treated cells (Figure 2B). Together these studies suggest that the microglial activation state induced by αSyn_agg_ may involve a functional interaction between NLRP3 and TXNIP in primary microglia.

### αSyn_agg_ Stimulated the Interaction Between TXNIP and NLRP3 in Mouse Primary Microglia

**Figure 3:**
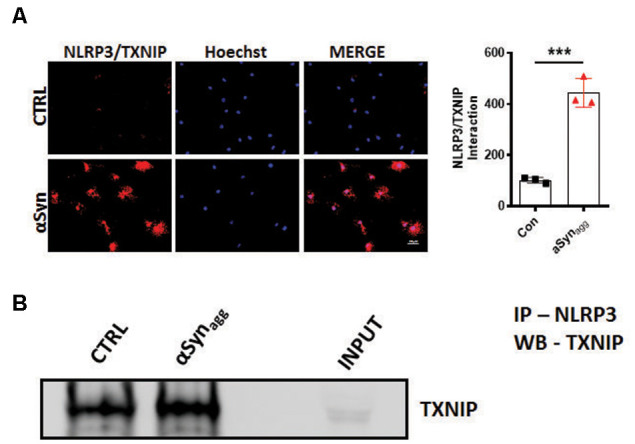
αSyn_agg_ promotes TXNIP interaction with NLRP3 in mouse primary microglia. **(A)** Representative immunofluorescence images of *in situ* proximity ligation assay (PLA) displaying interaction between TXNIP and NLRP3 in mouse primary microglia stimulated with αSyn_agg_ for 24 h. Quantification of PLA puncta reveals the maximal amount of PLA puncta in αSyn_agg_ stimulated mouse PMG. TXNIP-NLRP3 proximity ligation signal (red), and DAPI (blue). **(B)** Co-IP and WB analysis demonstrating the interactions between TXNIP and NLRP3 in αSyn_agg_ stimulated primary microglia. Quantification of TXNIP-NLRP3 interactions using densitometric scanning analysis. Cell lysates were prepared from mouse primary microglia stimulated with or without αSyn_agg_ for 24 h and subsequently immunoprecipitated (IP) with NLRP3 followed by immunoblotting with a TXNIP antibody. Data are shown as mean ± SEM. ***p < 0.001 vs. αSyn or controls using two-tailed *t*-test; *N* = 3).

Previous studies have identified mitoROS as a critical causative factor in promoting Trx disassociation from TXNIP, and its subsequent association with NLRP3, thereby leading to the generation of inflammasome activation markers (Zhou et al., 2010; Han et al., 2018). Therefore, we next assessed the interaction between NLRP3 and TXNIP using proximity ligation assay (PLA) in αSyn_agg_-stimulated cells. Immunofluorescence analysis revealed a significant interaction between endogenous TXNIP and NLRP3 proteins in αSyn_agg_-stimulated mouse primary microglia as compared to vehicle-treated cells (Figure 3A). We further validated our PLA findings using IP/IB analysis whereby a significant interaction between TXNIP and NLRP3 was evidenced in αSyn_agg_ stimulated mouse primary microglia as compared to controls (Figure 3B) suggesting that the TXNIP/NLRP3 association may at least in part trigger a reactive microglial activation state in the context of α-synucleinopathy.

### Salubrinal (SAL), an eIF2α Inhibitor, Mitigates αSyn_agg_-Induced Microglial Activation by Regulating TXNIP Expression, NLRP3 Inflammasome Activation, and Pro-inflammatory Cytokine Generation in Mouse Primary Microglia

**Figure 4:**
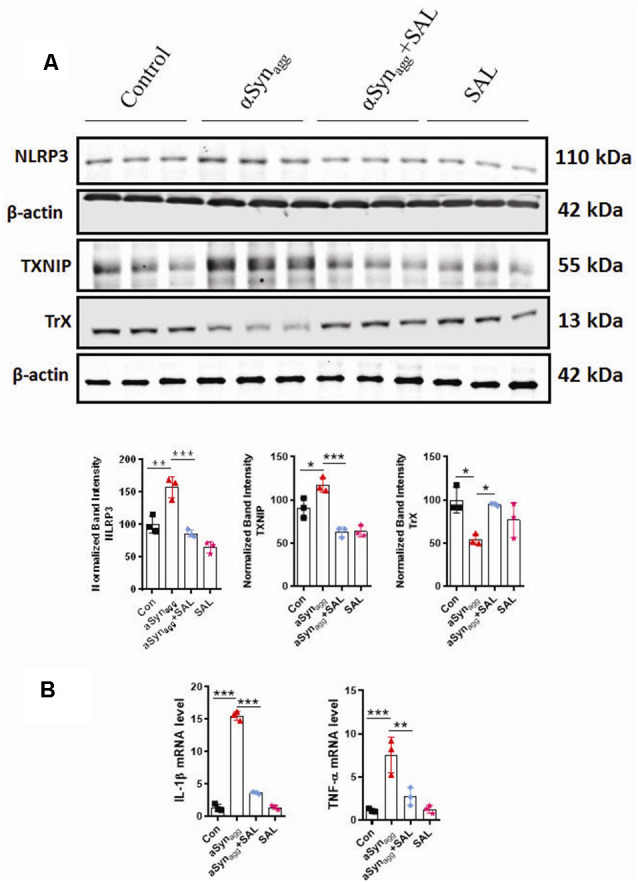
Salubrinal (SAL) attenuates the αSyn_agg_-induced activation of TXNIP, NLRP3 expression, and the release of pro-inflammatory cytokines in mouse primary microglia. Mouse primary microglia were pretreated with SAL (50 μM) for 3 h, followed by αSyn_agg_ for another 24 h. **(A)** Representative immunoblotting and densitometric quantification showing that SAL reduced the αSyn_agg_-induced upregulation of NLRP3, TXNIP, and concurrent downregulation of TrX. **(B)** Quantitative gene expression analysis (qRT-PCR) showing that SAL abolished the αSyn_agg_-induced mRNA expression of TNF-α, IL-1β in mouse primary microglia, 18S gene used for the normalizations. Data shown are the mean ± SEM from at least three independent experiments. Data were analyzed using one-way ANOVA followed by Bonferroni’s *post hoc* analysis. Asterisks (***p < 0.001, **p < 0.01 and *p ≤ 0.05) indicate significant differences between control and treatment groups.

Previous reports suggest that ERS is a critical regulator of the TXNIP/NLRP3 signaling axis and resultant inflammatory response in diverse experimental models of inflammation-related disorders (Chen X. et al., 2019; Feng and Zhang, 2019). Furthermore, pharmacological inhibition of ERS was found to afford neuroprotection in the A53T mouse model of PD (Boyce et al., 2005). However, the link between ERS and TXNIP/Trx, redoxisome dysfunction during an αSyn_agg_-induced microglial pro-inflammatory signaling event remains poorly characterized. We hypothesized that the TXNIP-Trx system is a critical intermediate that links ERS to microglia-mediated NLRP3 inflammasome activation subsequent to αSyn_agg_ stimulation. Therefore, we examined the contribution of ERS in TXNIP-Trx dysfunction utilizing SAL, which is a selective inhibitor of eIF2α and has been shown to exhibit anti-inflammatory effects in a rodent model of traumatic brain injury (Logsdon et al., 2016). We first examined whether SAL inhibited αSyn_agg_-induced dysregulation of the TXNIP/Trx system. Using mouse primary microglia, we first determined whether 50 μM of SAL (Huang et al., 2012) modulated the expression of ERS markers, including p-eIF2α, in αSyn_agg_-treated cells. Pretreatment with SAL increased αSyn_agg_-induced upregulation of p-eIF2α (data not shown). Next, we investigated the effect of SAL on αSyn_agg_-induced dysregulation of the TXNIP/Trx pathway in mouse primary microglia treated with αSyn_agg_. Our WB analysis revealed that SAL treatment attenuated αSyn_agg_-induced TXNIP upregulation while upregulating Trx expression (Figure 4A). Together, these results indicate that ERS blockade *via* SAL reduced the αSyn_agg_-induced dysregulation of the TXNIP/Trx signaling pathway which may, in turn, prevent the reactive microglial activation state. Emerging evidence suggests that ERS plays a critical role in TXNIP and resultant activation of the NLRP3 inflammasome (Lerner et al., 2012; Oslowski et al., 2012). However, to what extent ERS regulates NLRP3 inflammasome activation in response to αSyn_agg_ stimulation of mouse primary microglia remains poorly understood. Here we identified that NLRP3 inflammasome activation markers are activated in an ERS-dependent manner. Our immunoblot results showed that SAL attenuated the stimulatory effect of αSyn_agg_ on NLRP3 (Figure 4A), and its activation markers as evidenced by reduced pro-inflammatory cytokine mRNA expression of IL-1β and TNF-α (Figure 4B). These findings indicate that ERS blockade *via* SAL may in part ameliorate the αSyn_agg_-induced neurotoxic microglial activation state *via* inhibition of TXNIP/NLRP3 inflammasome axis activation in αSyn_agg_-stimulated primary microglia.

### Mito-Apocynin (MitoApo) Reduced NLRP3 Inflammasome Activation, ERS, and TXNIP/Trx Dysfunction in αSyn_agg_-Stimulated MMC Microglial Cells

**Figure 5:**
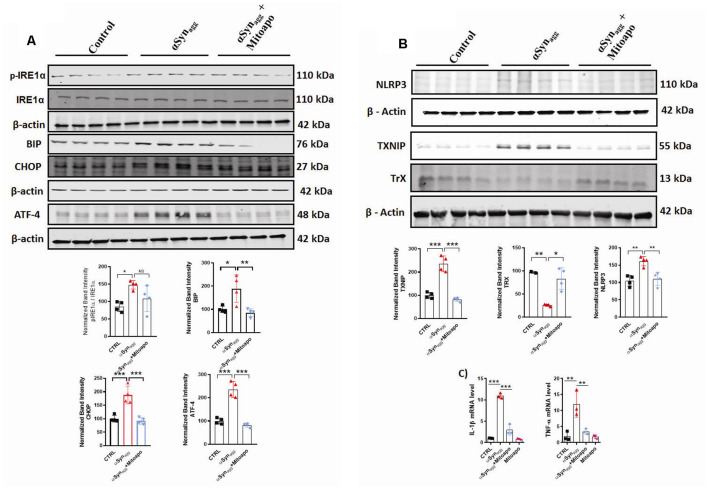
Inhibition of mitoROS *via* mito-apocynin (Mitoapo) attenuates αSyn_agg_-induced ERS, TXNIP/NLRP3 activation, and pro-inflammatory cytokine production in MMC microglial cells. MMCs were pretreated with Mito-Apo (10 μM) for 3 h, followed by a 24-h αSyn_agg_ treatment before being processed for WB and qRT-PCR analysis. **(A)** Representative immunoblot and densiometric quantification of protein expression showing that Mitoapo reduced the αSyn_agg_-induced upregulation of p-IRE1α, BIP, CHOP, and ATF-4. **(B)** Representative immunoblot and densitometric quantification of protein expression showing that Mitoapo reduced the αSyn_agg_-induced upregulation of TXNIP, NLRP3 while enhancing TrX expression levels. **(C)** qRT-PCR analyses showing mitoapo reduced the αSyn_agg_-induced increased generation of proinflamamtory cytokines TNF-α and IL-1β. Data shown are the mean ± SEM from at least three independent experiments. Data were analyzed using one-way ANOVA followed by Bonferroni’s *post hoc* analysis. Asterisks (***p < 0.001, **p < 0.01 and *p ≤ 0.05) indicate significant differences between control and treatment groups. NS: not significant.

Since αSyn_agg_ induces mitochondrial oxidative stress in primary microglia, next we explored the effects of (MitoApo), a mitochondrially targeted antioxidant on the αSyn_agg_-induced ERS and TXNIP/NLRP3 signaling cascade. Owing to the fact that mouse microglial cell line, MMC closely mimic neonatal primary microglia (Sarkar et al., 2020), we utilized these cells to determine the activation of the aforementioned microglial pro-inflammatory mediators in MMCs treated with αSyn_agg_ in the presence or absence of mitoapocynin. A previous report demonstrated that ER-mitochondria proximity promotes NLRP3 localization to mitochondria, triggering its activation (Shimada et al., 2012). Indeed, we previously demonstrated that the NLRP3 inflammasome translocated to the mitochondria in rotenone-stimulated microglial cells, which coincided with an elevated mitochondrial oxidative stress (Lawana et al., 2017). Based on these findings, we hypothesized that blocking mitochondrial oxidative stress *via* MitoApo would reduce ERS and the associated TXNIP/NLRP3 signaling pathway. To address this hypothesis, MMC microglial cells were treated with MitoApo (10 μM). We found that there was a significant upregulation of ERS markers including eIF2α, ATF-4, TXNIP, and NLRP3 protein expression, as well as pro-inflammatory cytokine mRNA expression (IL-1β and TNF-α) that was accompanied by an upregulation of Trx expression in MMC cells treated with αSyn_agg_, which was markedly reduced by treatment of mitoapocynin (Figures 5A–C). Collectively, these results indicate that MitoApo ameliorates the αSyn_agg_-induced ERS and TXNIP/NLRP3 signaling axis in a cell culture model of α-synucleinopathy.

### PKCδ Knockdown in MMC Microglial Cells, Ameliorated αSyn-Induced ERS as Well as TXNIP and NLRP3 Inflammasome Activation

**Figure 6:**
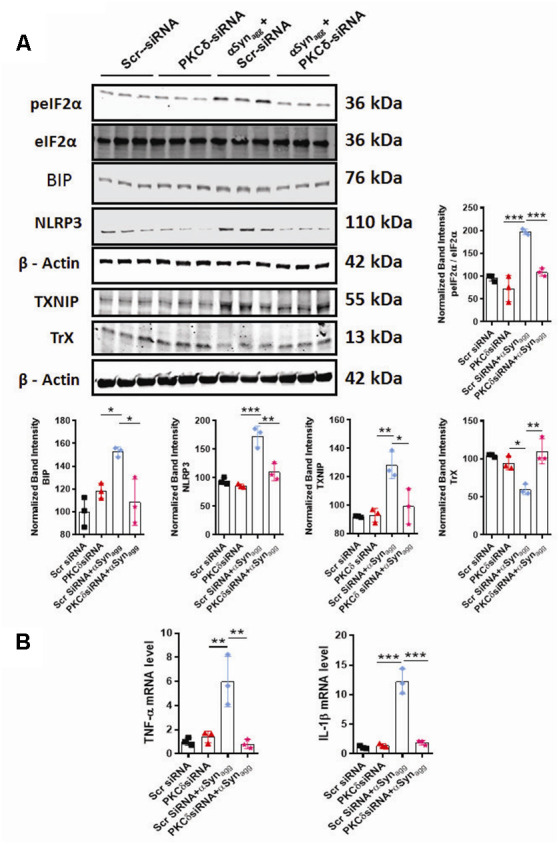
siRNA-mediated PKCδ knockdown alle*via*tes αSyn_agg_-induced ERS, TXNIP, and NLRP3 inflammasome activation in mouse microglial cells (MMCs). MMCs were transfected with scrambled siRNA (Scr siRNA) or PKCδ siRNA for 48 h and were then treated with αSyn_agg_ for another 24 h before being processed for Western blot and qRT-PCR analyses. **(A)** Representative immunoblot and densitometric quantification revealing that PKCδ-silencing diminished the αSyn_agg_-mediated activation of p-eIF2α, BIP, TXNIP, and NLRP3 and reversed the αSyn_agg_-induced suppression of TrX. Data shown are the mean ± SEM from at least three independent experiments. **(B)** qRT-qPCR analysis depicting that PKCδ gene depletion significantly attenuated the αSyn_agg_-induced mRNA expression of the pro-inflammatory markers IL-1β and TNF-α. Data shown are the mean ± SEM from at least three independent experiments. Data were analyzed using one-way ANOVA followed by Bonferroni’s *post hoc* analysis. Asterisks (***p < 0.001, **p < 0.01, and *p < 0.05) indicate significant differences between control and treatment groups.

We next determined whether siRNA-mediated knockdown of PKCδ altered the αSyn_agg_-induced microglial activation response. We have demonstrated that the redox-sensitive kinase PKCδ is linked to microglial NLRP3 inflammasome activation and IL-1β generation under oxidative stress conditions (Lawana et al., 2017; Panicker et al., 2019), raising the possibility that PKCδ may be involved in NLRP3 inflammasome activation *via* the ERS response. To determine whether or how PKCδ expression influence the αSyn_agg_-induced ERS-associated microglial activation response, MMC mouse microglial cells were transfected with either control siRNA or PKCδ siRNA and subsequently treated with αSyn_agg_. MMC microglial cells that were transfected with a small interfering RNA (siRNA) against PKCδ for 48 h displayed markedly reduced endogenous PKCδ levels (60–70%) as compared with scrambled siRNA-transfected cells (Supplementary Figure 2). We next evaluated the effects of RNAi-mediated knockdown of PKCδ on the αSyn_agg_-induced ER stress response in MMCs. Furthermore, as expected, downregulation of PKCδ remarkably repressed the αSyn_agg_-induced ER stress response as exemplified by reduced expression of BIP (Binding immunoglobulin protein), p-eIF2α in MMCs (Figure 6A).

Next, we investigated the impact of PKCδ knockdown on the expression of TXNIP and its binding partner Trx in αSyn_agg_-stimulated microglial cells using WB analysis. PKCδ downregulation dramatically decreased the expression of TXNIP while upregulating Trx expression in microglial cells treated with αSyn_agg_ (Figure 6A). Finally, we tested the causal relationship between PKCδ activation and NLRP3 inflammasome activation in microglial cells stimulated with αSyn_agg_. PKCδ knockdown in MMC microglial cells treated with αSyn_agg_ significantly reduced the expression of NLRP3 (Figure 6A) and mRNA expression of proinflammatory cytokines including TNF-α and IL-1β (Figure 6B). Taken together, these results demonstrate that downregulation of PKCδ reduces the αSyn_agg_-induced microglial activation response at least in part *via* amelioration of ERS and the TXNIP/NLRP3 signaling axis and the associated generation of proinflammatory cytokines in MMC microglial cells.

### TXNIP Downregulation Mitigated NLRP3 Inflammasome Activation Markers in Mouse Primary Microglia Stimulated With αSyn_agg_

**Figure 7:**
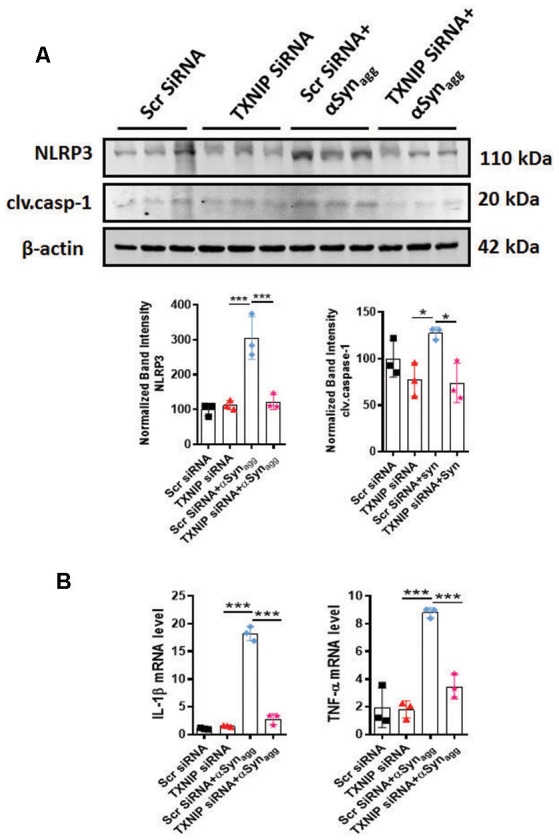
siRNA-mediated TXNIP knockdown mitigates NLRP3 inflammasome activation markers in primary microglial cells stimulated with αSyn_agg_. **(A)** Expression of caspase-1 and NLRP3 inflammasome activation marker in mouse PMG transfected with TXNIP siRNA for 48 followed by stimulation with αSyn_agg_ for another 24 h. Western blot analysis and quantification reveals inhibitory effects of TXNIP siRNA on αSyn_agg_-induced upregulation of NLRP3 and cleaved caspase-1 in mouse PMG as compared with scramble siRNA transfected cells. β-actin used as an internal control. **(B)** qRT-PCR analysis of IL-1β and TNF-α in mouse PMG transfected with or without TXNIP siRNA with or without stimulation with αSyn_agg_ for 24 h. TXNIP siRNA attenuated αSyn_agg_-induced upregulation of the aforementioned proinflammatory cytokine gene expression as compared to scramble transfected mouse PMG. Data shown are the mean ± SEM from at least three independent experiments. Significance is based on two-way ANOVA followed by a Bonferroni *post hoc* test. ***p < 0.001 and *p < 0.05 vs. controls.

Having demonstrated that TXNIP associates with NLRP3, we next determined whether TXNIP regulated NLRP3 inflammasome activation. Our approach was to assess NLRP3 activation markers since previous studies have demonstrated a positive association between TXNIP upregulation and activation and NLRP3 inflammasome activation markers (Tseng et al., 2016). Thus, in the initial set of experiments, we downregulated TXNIP expression using siRNA-mediated knockdown to determine its impact on NLRP3 inflammasome activation markers. Our WB analysis revealed a 65% knockdown efficiency (Supplementary Figure 3) of TXNIP that was accompanied by a marked reduction in the protein expression of NLRP3 inflammasome and cleaved caspase-1 expression (Figure 7A) in mouse primary microglia exposed to αSyn_agg_. In parallel studies, qPCR analysis revealed that TXNIP siRNA ameliorated αSyn_agg_-induced mRNA expression of IL-1β and TNF-α (Figure 7B). Our findings indicate a mechanistic link between TXNIP and the NLRP3 inflammasome activation in promoting a proinflammatory microglial phenotype and that downregulation of TXNIP ameliorates the proinflammatory microglial activation response in αSyn_agg_-stimulated mouse primary microglia.

### ERS Inhibition *via* SAL Decreased Indirect DAergic Neurotoxicity Mediated by αSyn_agg_ Stimulated Primary Microglia

**Figure 8:**
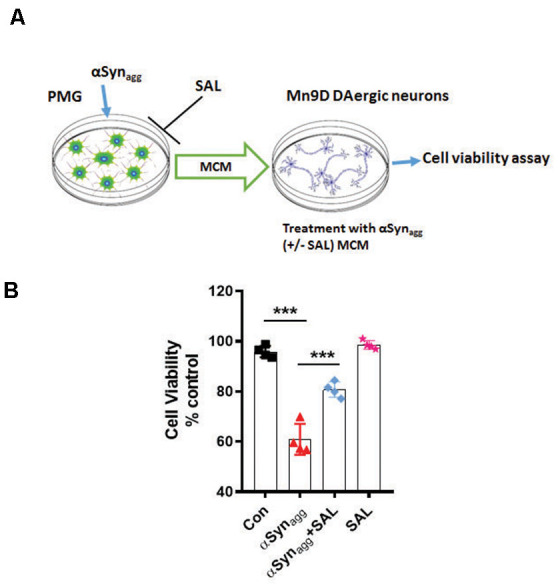
Salubrinal attenuates αSyn_agg_-stimulated microglia-conditioned media (MCM)-induced DAergic neurotoxicity. **(A)** Schematic diagram depicting the treatment of MN9D DAergic neuronal cells with MCM from αSyn_agg_-stimulated primary microglia. **(B)** SAL reduced αSyn_agg_ MCM-induced DAergic cell death. Mouse PMG was incubated with αSyn_agg_ for 24 h, and subsequently, MCM from αSyn_agg_-treated microglia with or without SAL was applied to MN9D DAergic neuronal cells for 12 h. At the end of the incubation period, an MTS assay was performed to determine the cell viability of MN9D DAergic neuronal cells. Data shown are the mean ± SEM from at least four independent experiments. Data were analyzed using one-way ANOVA followed by Bonferroni’s *post hoc* analysis. Asterisks (***p < 0.001) indicate significant differences between control and treatment groups.

To further test the influence of αSyn_agg_-induced microglial ERS on DAergic neuronal survival we treated primary microglia with αSyn_agg_ in the presence or absence of the ERS inhibitor SAL followed by 3-(4, 5-dimethylthiazol-2-yl)-2, 5-diphenyltetrazolium bromide (MTT) cell viability assay in MN9D DAergic neuronal cells treated with microglia-conditioned media (MCM) collected from αSyn_agg_-stimulated microglial cells treated with or without SAL. Mouse primary microglia were pretreated with SAL for 6 h and subsequently stimulated with αSyn_agg_ for 12 h, followed by washing and incubation with fresh cell culture media for another 24 h (Figure 8A). The resulting MCM was transferred to MN9D DAergic neuronal cells. After 18 h following MCM addition, cell viability was assessed using an MTT assay. The MCM collected from αSyn_agg_-stimulated mouse primary microglia increased MN9D DAergic cell death whilst this effect was markedly reduced in MN9D cells that were treated with MCM from SAL-pretreated, αSyn_agg_-stimulated microglial cells (SAL/αSyn_agg_-MCM; Figure 8B). These results are in line with previous studies showing that a neurotoxic microglial activation state exerts detrimental effects on DAergic neuronal integrity subsequent to the αSyn-induced microglial inflammatory response (Yun et al., 2018).

### Chronic Activation of ER Stress, TXNIP, and NLRP3 Inflammasome Activation Preceded Delayed Loss of TH+ Neurons in the Nigra of αSyn_PFF_-Inoculated Mice

**Figure 9:**
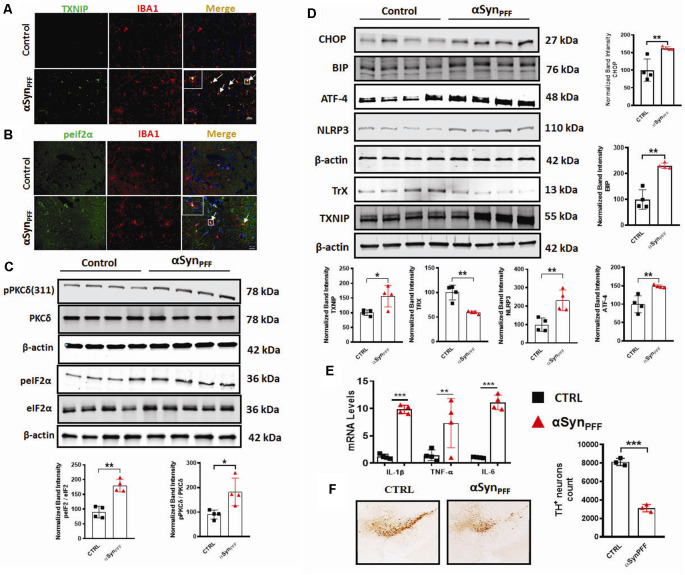
Chronic early activation of PKCδ, endoplasmic reticulum stress (ERS), and the TXNIP/NLRP3 signaling axis precedes the delayed tyrosine hydroxylase (TH) neuronal loss in the αSyn_PFF_ mouse model of PD. C57BL/6 mice were intrastriatally infused with either 2 μl of αSyn_PFF_ or saline (PBS) *via* stereotaxic injection. **(A,B)** Increased colocalization of ERS or inflammation-related marker in the nigral microglia of αSyn_PFF_ infused mice. Representative immunohistochemical images showing colocalization of either TXNIP or p-eIF2α within IBA-1 positive microglia in the nigra of αSyn_PFF_ infused mice as compared to the PBS-injected mice. Nigral brain sections were double immunolabeled for p-eIF2α/IBA-1 or TXNIP/IBA-1, Nuclei were counterstained for hoechest. **(C)** Western blots showing increased expression of PKCδ and p-eIF2α in the nigra of αSyn_PFF_ inoculated mice. **(D)** Representative immunoblots showing that increased expression of CHOP, BIP, ATF-4, NLRP3, TXNIP, and downregulation of TrX in the SNpc of αSyn_PFF_ inoculated mice. Quantification of the relative expression of the aforementioned proteins at 60 dpi. **(E)** qRT-PCR analysis showing increased expression of IL-6, TNF-α, and IL-1β in the striata of αSyn_PFF_-infused mice as compared to PBS-infused mice. **(F)** Representative photomicrographs of diaminobenzidine (DAB) immunostaining of TH in coronal midbrain sections of SN from perfused mouse brains at 180 dpi showing a dramatic reduction of TH-positive neurons in αSyn_PFF_-treated mice vs. PBS-treated mice. Data are the mean ± SEM; *n* = 4 independent mice. Data shown are the mean ± SEM from at least three independent experiments. Asterisks (***p < 0.001, **p < 0.01 and *p ≤ 0.05) indicate significant differences between control and treatment groups. NS: not significant.

Our *in vitro* studies revealed that treatment of primary microglia induced PKCδ activation with an accompanying induction of the ERS-mediated TXNIP/NLRP3 signaling axis. Next, we sought to investigate the aforementioned signaling events in the αSyn_PFF_ mouse model of sporadic PD that reproduces several key PD pathological correlates including αSyn pathology, loss of DAergic neurons, and the microglial activation response *in vivo*. In this model, αSyn_PFF_ seeds are taken up by the striatum and transmitted to the nigra in a retrograde fashion *via* the nigrostriatal pathway where they function as a template and promote the seeding of endogenous murine αSyn to accumulate into misfolded phosphorylated pathological aggregates (Luk et al., 2012; Earls et al., 2019). Although microglia has been implicated in neuroinflammation and associated nigral TH neuronal loss (Duffy et al., 2018). To date, there is no published literature detailing TXNIP nor eIF2α-expression within microglia in the context of synucleinopathy. Therefore, we used specific markers of ERS and inflammation to further support the role of microglial ERS in PD like pathology. To this end, we performed double immunolabeling for p-eIF2α or TXNIP a in nigral brain section from αSyn_PFF_ and PBS (control) infused mice. A previous study reported that TXNIP colocalizes with microglia after subarachnoid hemorrhage (SAH; Zhao et al., 2017a). As expected, control mice displayed scant expression of eIF2α or TXNIP. In contrast, αSyn_PFF_-infused mice display considerable colocalization of eIF2α and TXNIP within IBA-1-positive microglia (Figures 9A,B). Thus, our results demonstrate that both p-eIF2α and TXNIP colocalized with microglia in the substantia nigra of αSyn_PFF_-infused mice, suggesting that these inflammatory markers may aggravate nigral DAergic neurotoxicity by triggering pro-inflammatory signaling events. Moreover, we cannot exclude the possibility that activation of the aforementioned proinflammatory factors in other CNS cell types might contribute to the nigral DAergic neurotoxicity. Additionally, consistent with our *in vitro* results, our WB analysis further confirmed that αSyn_PFF_ intrastriatal infusion significantly upregulated the ERS markers p-eIF2α, CHOP (C/EBP Homologous Protein), BIP, and ATF-4 (Figures 9C,D) in the SNpc, which positively correlated with TXNIP upregulation and the associated downregulation of Trx levels as compared to PBS-infused mice (Figure 9A). Likewise, this effect was accompanied by PKCδ activation and upregulation of the NLRP3 inflammasome in the SNpc that was associated with enhanced generation of proinflammatory cytokine mRNA levels including Il-1β, TNF-α, and IL-6 in the striatum as compared to PBS-infused mice at 60 dpi (Figure 9E). Next, we determined whether TH neuronal loss occurs during the latter part of the disease (180 dpi) *in vivo* as demonstrated previously (Duffy et al., 2018). As expected, delayed TH^+^ neuron loss in the SN of αSyn_PFF_-infused mice was evidenced at 180 dpi as compared to PBS infused mice as determined by unbiased stereological analysis (Figure 9F). These results are consistent with our hypothesis that irremediable microglial ERS increases the susceptibility of nigral DAergic neurons to neurodegeneration in the context of α-synucleinopathy concomitant with the induction of TXNIP/NLRP3 mediated proinflammatory signaling events.

## Discussion

Microglia-mediated neuroinflammation has been linked to PD pathogenesis (Hirsch et al., 2012). Moreover, the identification of elevated inflammatory markers in postmortem PD brains (Ferrari et al., 2006; McCoy et al., 2006) and the discovery of PD risk genes that serve as risk factors of both sporadic and familial PD (Zimprich et al., 2004; Satake et al., 2009; Simón-Sánchez et al., 2009) further supports the link between neuroimmune dysfunction and PD pathogenesis. Intriguingly, Tokuda et al., have demonstrated elevated αSyn oligomer levels and an increased oligomer/total αSyn ratio in the CSF of PD patients, suggesting a pivotal role of this protein in the etiology of PD (Tokuda et al., 2010). Lewy bodies encompassing aggregated aSyn have been hypothesized to play a critical role in the pathogenesis of synucleinopathies (Larks, 1958).

Emerging evidence indicates that fibrillar αSyn can act as seeds to promote misfolding and aggregation of endogenous αSyn, in both cellular (Volpicelli-Daley et al., 2014) and animal models of PD (Luk and Lee, 2014) in the absence of αSyn overexpression. Intriguingly, a recent report suggested that the process of Lewy body formation involving interactions between membranous organelles and αSyn aggregates that contain αSyn competent seeding species, most likely in an rearranged fibrillar conformation is a major driver of neurodegeneration (Mahul-Mellier et al., 2020). This study suggested that the processes associated with LB formation and subsequent maturation are critical drivers of αSyn mediated neurotoxicity than αSyn fibril formation. Moreover, recent studies have shown that exogenously added aggregated αSyn can trigger misfolding and aggregation of endogenous αSyn, in cell culture (Panicker et al., 2019) while αSyn_PFF_ intrastriatal inoculation in rodents causes inclusion formation and associated nigral DAergic pathology within weeks post-injection (Paumier et al., 2015). Moreover, previous studies have shown that extracellular αSyn aggregates can induce the microglial activation response which, in turn, promotes the propagation of aggregated αSyn within interconnected brain regions (Boza-Serrano et al., 2014; Duffy et al., 2018; Yun et al., 2018) suggesting that microglia are major drivers of neuroinflammation and associated PD-like pathology. Given the positive association between αSyn pathology and microglial NLRP3 inflammasome activation in experimental Parkinsonism (Gordon et al., 2018; Panicker et al., 2019), we investigated the contribution of mitochondrial oxidative stress and ERS in the αSyn_agg_-induced NLRP3-dependent microglial activation response. In this study, we provide compelling evidence that αSyn_agg_ elicits the microglial activation response in part *via* the induction of the PKCδ-dependent ERS-mediated TXNIP/NLRP3 signaling axis. In agreement with these results, prior work from our lab and others suggest that aggregated αSyn induced neurotoxic microglial activation state may exert deleterious effects on DAergic neuronal survival (Duffy et al., 2018; Gordon et al., 2018; Yun et al., 2018). Importantly, blockade of mitoROS *via* MitoApo attenuates ERS and the associated TXNIP/NLRP3 signaling axis in microglial cells. Likewise, the eIF2α inhibitor SAL not only attenuated αSyn_agg_-induced microglial TXNIP/NLRP3 signaling but also indirect, microglia-mediated DAergic neurotoxicity. More interestingly, PKCδ activation correlated positively with the enhanced microglial ERS and TXNIP/NLRP3 signaling axis and was found to precede TH neuronal loss in the nigra of mice that received an intrastriatal injection of αSyn_PFF_, implicating the interdependency between microglial PKCδ activation and induction of the TXNIP/NLRP3 signaling axis in α-synucleinopathy.

Given the central role of chronic neuroinflammation in promoting αSyn deposition in several synucleinopathy animal models (Choi et al., 2009; Frank-Cannon et al., 2009; Gao et al., 2011), deciphering the molecular basis of the αSyn_agg_-induced microglial activation response is critical. For this purpose, we analyzed the factors regulating NLRP3 inflammasome activation subsequent to the αSyn-induced microglial activation response. Exogenous αSyn_agg_ may interact with microglia *via* two different mechanisms. On the one hand, it may interact with CD36, class B scavenger receptor leading to the internalization of αSyn_agg_ by microglia leading to the microglial activation response *via* the Fyn-mediated NLRP3 inflammasome activation mechanism (Panicker et al., 2019). On the other hand, caspase-1-mediated truncation and aggregation of αSyn has been shown to promote uptake into microglial cells *via* TLR2 and TLR4 subsequently activating the NLRP3 inflammasome (Stefanova et al., 2011; Kim et al., 2013; Gustot et al., 2015; Chatterjee et al., 2020; Fan et al., 2020). Consistent with the former report, we observed that αSyn_agg_ was internalized within primary microglia suggesting that it may serve as a DAMP (Disease Associated Molecular Pattern) signal, thereby leading to NLRP3 inflammasome dependent, caspase-1-mediated IL-1β production (Codolo et al., 2013). Alternatively, recent studies from our group and others have demonstrated that many different types of cellular stress, including oxidative stressors/mitochondrial toxicants (Kim et al., 2014; Abais et al., 2015; Chen et al., 2015) or ERS inducers (Menu et al., 2012; Wali et al., 2014; Lebeaupin et al., 2015; Wang et al., 2017), may also promote NLRP3 inflammasome activation. Consistent with these findings, a positive association between robust mitoROS generation and NLRP3 inflammasome activation markers was evidenced in αSyn_agg_-stimulated primary microglia. Importantly, Thioredoxin-interacting protein (TXNIP) a binding partner of thioredoxin (Trx) that inhibits the reducing activity of Trx through their disulfide exchange (Yoshihara et al., 2014), has been linked to NLRP3 inflammasome activation *via* increased oxidative stress (Chen et al., 2009; Oslowski et al., 2012). In line with these findings, we found that protein expression of TXNIP was upregulated while Trx expression was downregulated in αSyn_agg_-stimulated primary microglia. Under divergent exogenous stress, TXNIP has been shown to bind to Trx thus disturbing the redox homeostasis, leading to ROS generation and resultant NLRP3 inflammasome activation, *via* a caspase-1-dependent IL-1β secretion mechanism which might be partially attributed to TXNIP interaction with Trx (Yoshihara et al., 2014; Zhao et al., 2017a). The significance of this interaction is further supported with data from αSyn_agg_ stimulated primary microglia demonstrating an interaction between TXNIP and the NLRP3 inflammasome that positively correlated with proinflammatory cytokine generation including IL-1β (Chen et al., 2009; Oslowski et al., 2012). Conversely, siRNA-mediated TXNIP knockdown attenuated NLRP3 inflammasome upregulation and proinflammatory cytokine generation including TNF-α, IL-1β. Additionally, we also demonstrated that inhibition of ERS *via* salubrinal also attenuated TXNIP through the upregulation of anti-oxidant protein Trx in αSyn_agg_-stimulated microglial cells. Indeed, a previous study demonstrated that SAL treatment attenuated oxidative stress and neuroinflammation in a traumatic brain injury (TBI) rodent model further highlighting the pivotal role of ERS in neuroinflammation-related neurological disorders (Logsdon et al., 2016). Thus, the ability of TXNIP to interact with the NLRP3 inflammasome and its subsequent activation may serve as a critical regulator of microglial activation in response to αSyn_agg_. While further studies are required, to demonstrate a direct contribution of NLRP3/TXNIP interaction to PD-like pathology, these studies raise the possibility that TXNIP and the NLRP3 inflammasome may be subject to regulation by both mitoROS and ERS and that the TXNIP/NLRP3 signaling axis may represent a feed-forward loop contributing to the enhancement of the αSyn_agg_-induced neurotoxic microglial activation state. Despite this, the contribution of microglial activation to the secretion of neurotrophic factors, such as brain-derived neurotrophic factor (Trang et al., 2011), cannot be entirely ruled out.

ERS contributes to the initiation and progression of numerous immune disorders including type 2 diabetes, obesity, atherosclerosis, and neurodegenerative diseases (Oakes and Papa, 2015; Cao et al., 2016; Grootjans et al., 2016; Tao et al., 2018). The link between neuronal ERS and α-synucleinopathy has been intensely investigated in genetic models of PD following overexpression of αSyn (Colla, 2019). For example, toxicity associated with WT, A53T mutant, or C-terminal truncated αSyn was found to positively correlate with ERS and UPR activation (Cooper et al., 2006; Bellucci et al., 2011; Heman-Ackah et al., 2017; Karim et al., 2020). Moreover, treatment with ERS inhibitor salubrinal, afforded protection against A53T αSyn-induced cell death indicating that ERS is a central contributor to DAergic cell death (Smith et al., 2005). Despite this, the mechanisms by which aberrant ERS contribute to the αSyn_agg_-induced microglial activation response remains poorly understood. Herein, we show that ERS markers, including p-eIF2α, ATF-4, p-IRE1α, BIP, are elevated in αSyn_agg_-stimulated primary microglia concomitant with the TXNIP/NLRP3 signaling markers and that SAL not only attenuated ERS but also the TXNIP/NLRP3 signaling axis and proinflammatory cytokine generation in line with previous reports (Oslowski et al., 2012; Logsdon et al., 2016; Lebeaupin et al., 2018). Importantly, SAL inhibited microglia-mediated indirect DAergic neurotoxicity, suggesting that ERS can promote a deleterious microglial neurodegenerative phenotype eventually contributing to the nigral DAergic neurodegenerative process *in vivo*, suggesting that microglial NLRP3 and TXNIP may represent critical pathological correlates in PD pathogenesis and other synucleinopathies.

Both mitochondria-mediated oxidative stress and neuroinflammation have been identified as key pathological correlates in the pathophysiology of PD (Beal, 2003; Rocha et al., 2018). Although mitochondrial dysfunction and ERS have been established as significant contributors to DAergic neuropathology (Chen C. et al., 2019), the impact of mitochondrial oxidative stress on ERS and associated microglia-mediated neuroinflammation remains poorly characterized. The generation of ROS *via* mitochondria has been linked to oxidative damage-induced pathologies including PD (Gao et al., 2008; Murphy, 2009; Dias et al., 2013). Our current study also revealed pronounced mitochondrial dysfunction characterized by robust mitoROS generation and an accompanying loss of MMP in αSyn-stimulated microglial cells. Moreover, we demonstrated that mitochondrial dysfunction preceded ERS in αSyn_agg_-stimulated primary microglia, suggesting that mitochondrial oxidative stress acts as an upstream regulator of ERS and the TXNIP/NLRP3 inflammasome signaling axis. Indeed, treatment with the mitochondria-targeted antioxidant MitoApo attenuated ERS markers, including ATF-4 and p-IRE-1α, with an accompanying decreased expression of NLRP3 markers and TXNIP expression, as well as decreased proinflammatory cytokine generation in αSyn_agg_-stimulated microglial cells. Our results suggest that the microglial inflammatory modulation triggered by mitochondrial oxidative stress is likely to promote the induction of ERS-dependent proinflammatory cell signaling events in synucleinopathies.

PKCδ-mediated cell signaling events occur in a cell type-specific and stimulus-specific manner. For example, recent findings from our lab and others have demonstrated that post-translational modification of PKCδ *via* a phosphorylation-dependent mechanism is associated with the Parkinsonian toxin-induced microglial activation response (Gordon et al., 2012; Panicker et al., 2015, 2019). Moreover, studies from our lab demonstrated that PKCδ is elevated in the microglia of postmortem PD brains and in animal models of PD (Gordon et al., 2016). Likewise, a similar increase in PKCδ was evidenced in human postmortem AD brains (Gordon et al., 2016b; Du et al., 2018). Despite this, the exact mechanism by which PKCδ contributes to the microglial activation response in the context of α-synucleinopathy remains undefined. Therefore, we hypothesized that PKCδ may have other effects, including the regulation of ERS-associated inflammatory signaling events in the microglia. Our results demonstrate that PKCδ is activated *via* a phosphorylation-dependent mechanism in αSyn_agg_-treated microglial cells (as assessed *via* a time-dependent increase in phosphorylation of PKCδ at Tyr412 residue), which closely paralleled upregulation of ERS markers including ATF4, p-eIF2α, p-IRE1α and pro-inflammatory cytokine generation including IL-6, and TNF-α with concurrent upregulation of the NLRP3 inflammasome activation markers (cleaved caspase-1 and IL-1β) and TXNIP expression. Conversely, we found that siRNA-mediated knockdown of PKCδ attenuated the αSyn_agg_-induced ERS response-mediated NLRP3/TXNIP signaling cascade, highlighting the regulatory effects of PKCδ on ERS and the associated proinflammatory response in microglial cells. Furthermore, using a mouse model of α-synucleinopathy known to recapitulate key pathological correlates of synucleinopathies, including the microglial activation response and DAergic neuropathology, we show that αSyn_PFF_ injection into the dorsal striatum resulted in pronounced PKCδ activation and ERS that was accompanied by upregulation of the NLRP3/TXNIP signaling axis in the nigra at 60 dpi, which represents an anatomically connected brain region. Therefore, aggregated αSyn may activate microglial PKCδ-dependent ERS-mediated activation of the TXNIP/NLRP3 signaling axis during the early stages of the pathology in the substantia nigra of αSyn_PFF_-inoculated mice as compared to controls. Indeed, another study demonstrated the occurrence of pronounced microglial activation in the nigra several months prior to expression of nigral DAergic neurotoxicity (Duffy et al., 2018; Patterson et al., 2019). Moreover, perturbation of protein degradation machinery could lead to an increased protein load in the ER, thus making the cell more vulnerable to ERS. Indeed, αSyn aggregation was found to result in protein accumulation in the ER followed by induction of UPR and the resulting cell death in an *in vitro* cell culture model of PD (Cooper et al., 2006). In line with this finding, early induction of the nigral microglial ERS/TXNIP signaling cascade was found to precede significant TH neuronal loss at 180 dpi. Moreover, injection of αSyn_PFF_ into the striatum was also found to result in increased mRNA expression levels of TNF-α, IL-1b, IL-6 in this brain region. Importantly, we previously reported that proinflammatory cytokine such as TNF-α acting *via* TNFR1 induced PKCδ-mediated apoptotic death of DAergic neurons (Gordon et al., 2012). Thus, our observations raise the possibility that initial activation of the unfolded protein response (UPR) in α-synucleinopathy might have a neuroprotective role to compensate for the accumulation of misfolded proteins; however, persistent ERS may overwhelm the cellular antioxidant machinery *via* PKCδ and TXNIP upregulation eventually leading to the loss of nigral DAergic neurons. Thus, ERS and TXNIP are likely to play a causative role in nigral DAergic neuronal death in response to αSyn_PFF_. Accordingly, increased TXNIP expression has been demonstrated in the hippocampus and cortex of the APP/PS1 transgenic mouse model of AD as an early disease phenotype (Wang Y. et al., 2019). Alternatively, ERS and TXNIP can also occur in other immune cell types such as astrocytes as well as other cells in the CNS further exaggerating nigral DAergic neurodegeneration, which requires further exploration. Given that males are at a higher risk of developing PD than women (Cerri et al., 2019) we utilized male mice in these studies. In light of the evidence demonstrating sex differences in inflammasome activation, we cannot rule out that different pathogenic mechanisms might be involved in the difference between men and women in expressing PD-related clinical correlates (Zhang et al., 2020).

**Figure 10:**
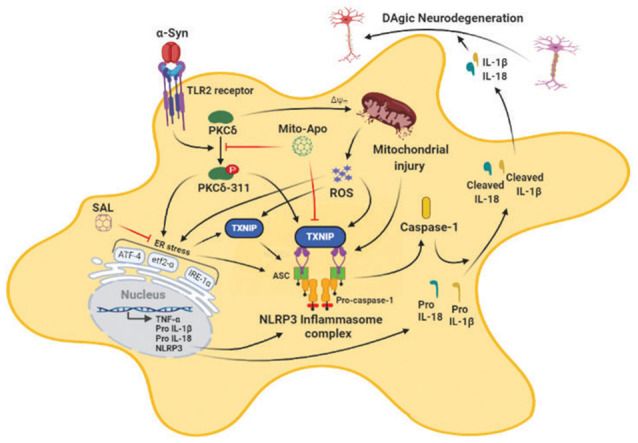
Schematic representation of the role of mitoROS-PKCδ-mediated endoplasmic reticulum stress (ERS)-dependent induction of the TXNIP/NLRP3 signaling axis in response to the αSyn_agg_-induced reactive microglial activation state. The treatment of microglial cells with αSyn_agg_ triggers mitoROS and PKCδ activation resulting in ERS-dependent activation of proinflamamtory signaling events including the TXNIP/NLRP3 activation response. Both mitochondria-driven oxidative stress and PKCδ activation contribute to the αSyn_agg_-induced reactive microglial activation state *via* ERS through a feed-forward mechanism. An association between TXNIP and NLRP3 may partly explain the NLRP3 inflammasome-associated innate immune response. Moreover, αSyn_agg_-induced microglial ERS causes indirect DAergic neurotoxicity. Importantly, Mitoapo and PKCδ-silencing suppress ERS and the associated TXNIP/NLRP3 signaling axis in response to αSyn_agg_, thereby attenuating the reactive microglial activation state. Finally, salubrinal (SAL) attenuated the indirect DAergic neurotoxicity elicited by αSyn_agg_, further highlighting the pivotal role of ERS in the expression of a neurodegenerative microglial phenotype and associated DAergic neurotoxicity.

Overall, our current findings coupled with other recent observations (Panicker et al., 2015; Gordon et al., 2016b), suggest that PKCδ and mitochondrial oxidative stress act as key regulators of ERs and TXNIP/NLRP3 inflammasome activation and support the hypothesis that PKCδ activation may drive the proinflammatory activation of microglia in response to αSyn_agg_ along with ERS and mitochondrial oxidative stress. The pathological significance of the aforementioned signaling pathways is further illustrated by the fact that *in vitro* PKCδ siRNA mediated knockdown, as well as pharmacological inhibition of mitochondrial oxidative stress and ERS, ameliorates the microglial activation response subsequent to stimulation of microglial cells with αSyn_agg_. Finally, our study highlights that intrastriatal αSyn_PFF_ injection, triggers microglial ERS and upregulation of the TXNIP/NLRP3 signaling axis are evidenced in the SN prior to late-stage nigral DAergic neurodegeneration providing further support for the significance of early induction of microglial ERS and TXNIP/NLRP3 signaling as an upstream instigating factor promoting PD-like pathology. Therefore, our studies unravel a previously unknown mechanism linking PKCδ, ERS, and the TXNIP/NLRP3 signaling axis to the microglial activation response in the context of α-synucleinopathy (Figure 10). Thus, therapeutic targeting of TXNIP and NLRP3 may represent a novel disease-modifying therapy for the treatment of PD.

## Data Availability Statement

The original contributions presented in the study are included in the article/Supplementary Material, further inquiries can be directed to the corresponding author.

## Ethics Statement

The animal study was reviewed and approved by Institutional Animal Care and Use Committee (IACUC) at Iowa State University, Ames, IA, USA.

## Author Contributions

MS, AGK, and AK created the research design and conceptualized the study. MS, BP, GZ, HJ, and VA developed the methodologies detailed in the manuscript. MS, BP, AB-C, MH, NK, SM, VA, AGK, and AK conducted the experiments and analyzed the experimental results. MS, AGK, and AK wrote the original draft of the manuscript. MS, GZ, HJ, VA, AGK, and AK participated in manuscript discussions, and in the review and editing of the manuscript. AK and AGK secured funding and provided resources to successfully complete the proposed experiments. All authors have read and agreed to the published version of the manuscript. All authors contributed to the article and approved the submitted version.

## Conflict of Interest

AGK and VA have an equity interest in PK Biosciences Corporation located in Ames, IA. AGK also has an equity interest in Probiome Therapeutics located in Ames, IA. The terms of this arrangement have been reviewed and approved by Iowa State University in accordance with its conflict-of-interest policies. The remaining authors declare that the research was conducted in the absence of any commercial or financial relationships that could be construed as a potential conflict of interest.
