# Supplementary material for: PKC Delta Activation Promotes Endoplasmic Reticulum Stress (ERS) and NLR Family Pyrin Domain-Containing 3 (NLRP3) Inflammasome Activation Subsequent to Asynuclein-Induced Microglial Activation: Involvement of Thioredoxin-Interacting Protein (TXNIP)/Thioredoxin (Trx) Redoxisome Pathway
Source: Front Aging Neurosci. 2021 Jul 2;13:661505. doi: 10.3389/fnagi.2021.661505 (PMC8283807; doi:10.3389/fnagi.2021.661505)
Supplement: Supplementary file 1 [file Data_Sheet_1.docx]

Supplementary Material

**B**

**A**

**Supplementary Fig 1.** (A). Mouse PMG cells were treated with αSyn_agg_ (1µM) for increasing time points (6 h, 12 h, 18 h, and 24 h) and then assayed for the cell viability. αSyn_agg_ treatment failed to induce cell death at any of the indicated time points in mouse PMG cells. (B) Nitric oxide levels in the supernatant were determined using the Griess reagent. αSyn_agg_ induced a time dependent increase in the level of nitric oxide generation. Data shown are Mean ± SEM from at least 3 independent experiments**.** Data were analyzed using one-way ANOVA followed by Bonferroni’s post hoc analysis. Asterisks (*** p < 0.001, ** p < 0.01 and * p ≤ 0.05) indicate significant differences between control and treatment groups.


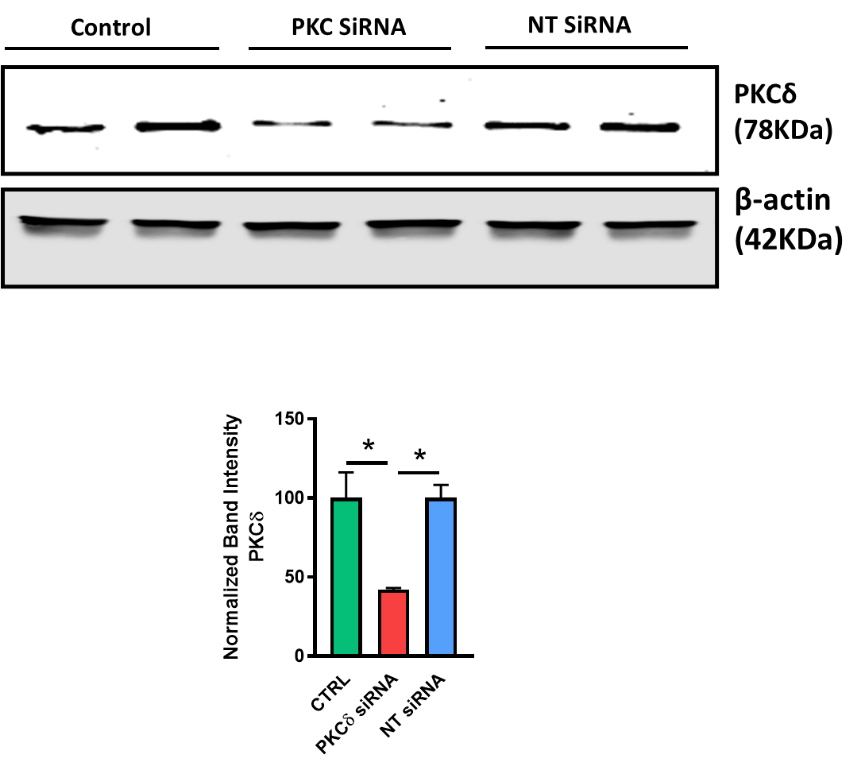

**Supplementary Fig 2.** Mouse microglia cells were transfected with scrambled siRNA or PKCδ siRNA and incubated for 48h. The efficiency of PKCδ knock down were analyzed using WB analysis. Quantification of immunoblots using densitometric analysis shows that siRNA-mediated gene silencing of PKCδ resulted in marked reduction in the endogenous PKCδ levels (60-70%) in mouse microglia cells (MMCs). Data shown are Mean ± SEM from at least 3 independent experiments. Data were analyzed using one-way ANOVA followed by Bonferroni’s post hoc analysis. Asterisks (*** p < 0.001, ** p < 0.01 and * p ≤ 0.05) indicate significant differences between control and treatment groups.


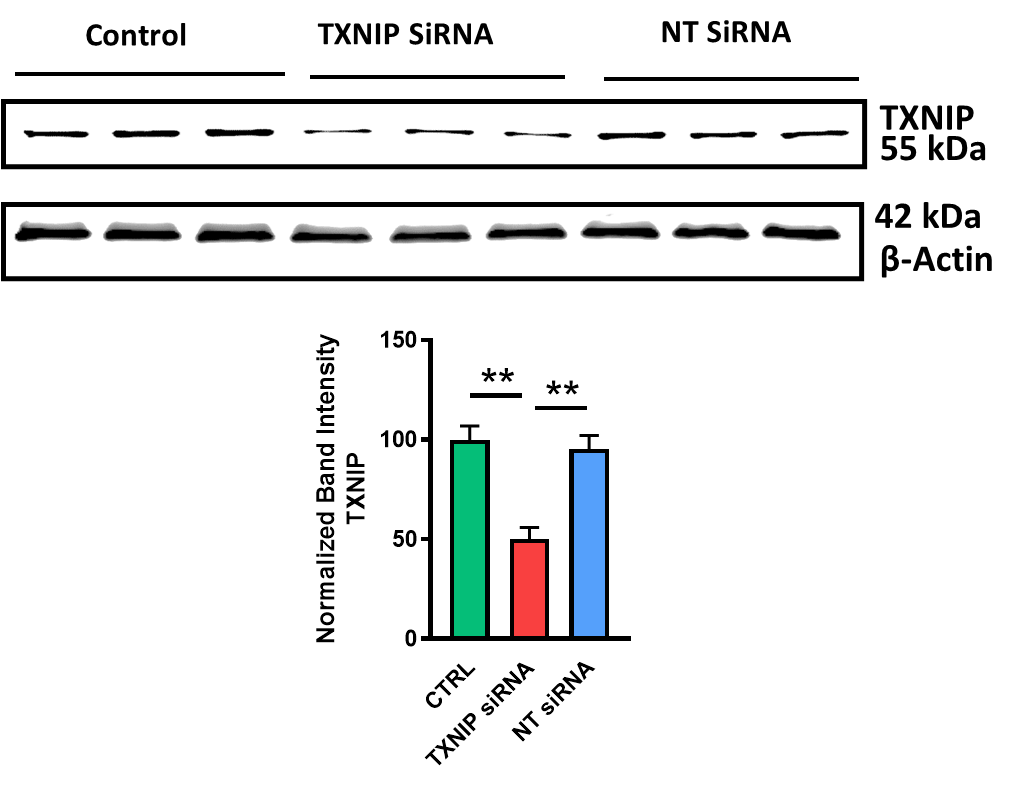

**Supplementary Fig 3.** Mouse microglia cells were transfected with scrambled scramble siRNA or TXNIP siRNA and incubated for 48 h. The efficiency of TXNIP knock down was analysed using WB analysis. Quantification of immunoblots reveal that siRNA-mediated gene silencing of TXNIP resulted in marked reduction in the endogenous TXNIP levels (60-65%) in mouse microglia cells. Data shown are Mean ± SEM from at least 3 independent experiments Data were analyzed using one-way ANOVA followed by Bonferroni’s post hoc analysis. Asterisks (*** p < 0.001, ** p < 0.01 and * p ≤ 0.05) indicate significant differences between control and treatment groups.

**
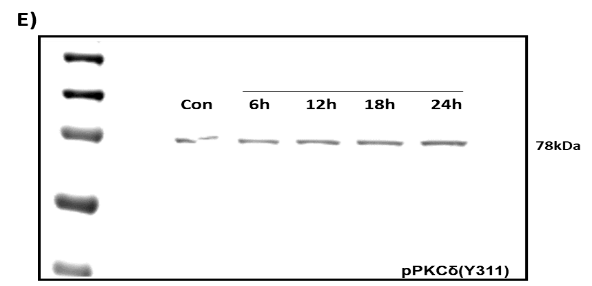

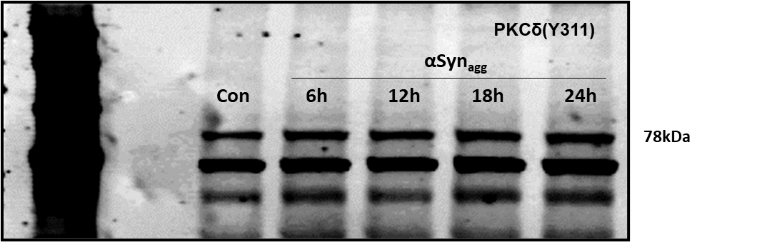
**

**
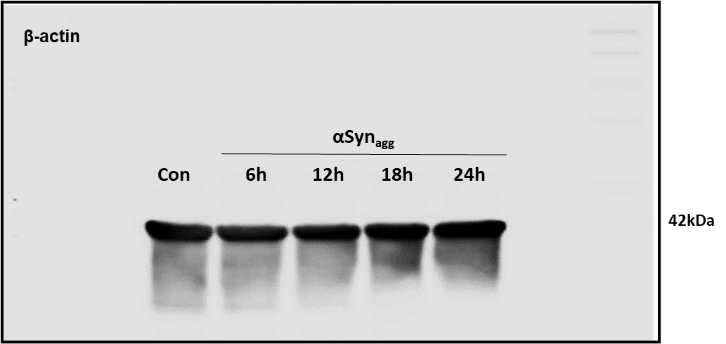

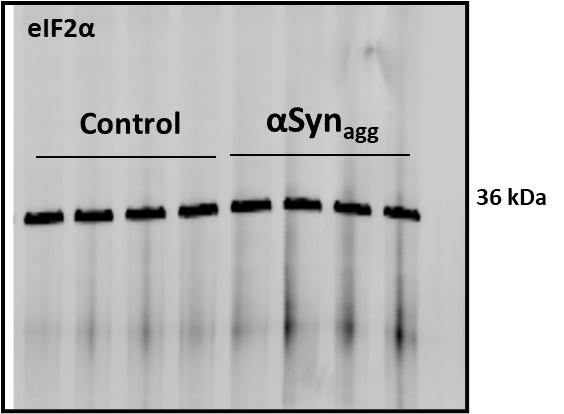
**

**
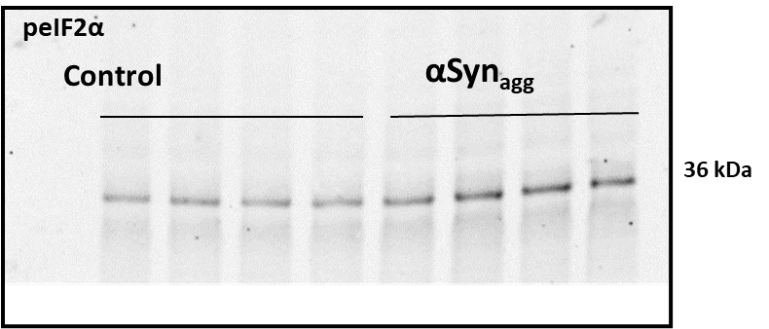

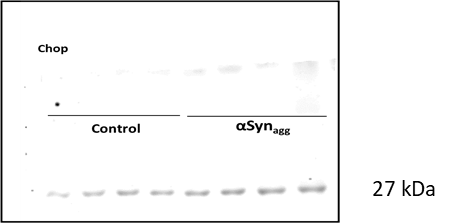
**

**
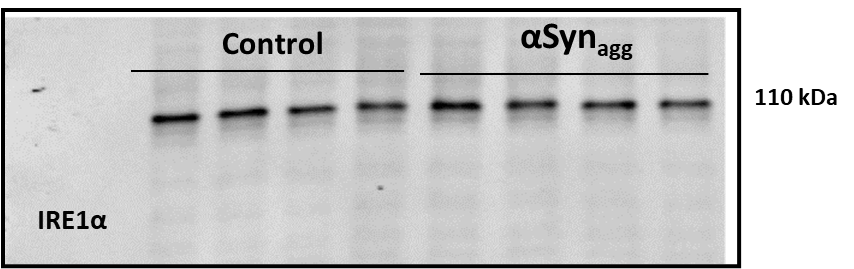

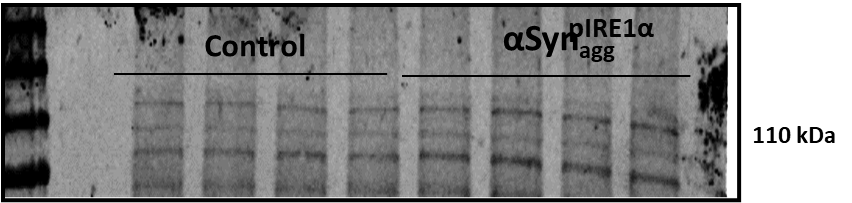
**

**
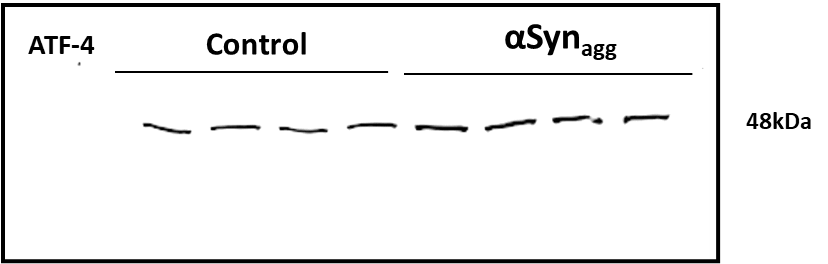

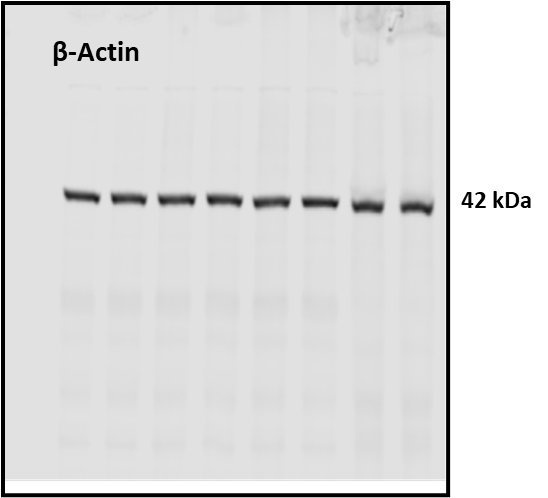
**

**Full blot images of Fig. 1**

**
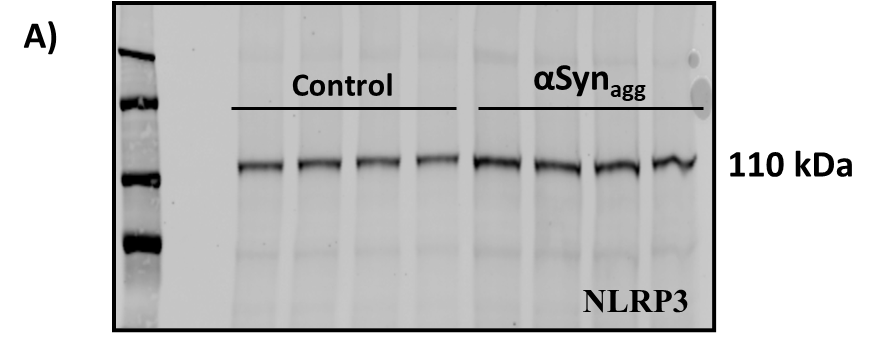

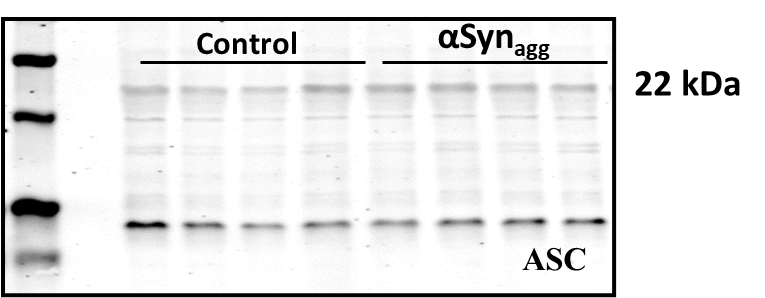
**

**
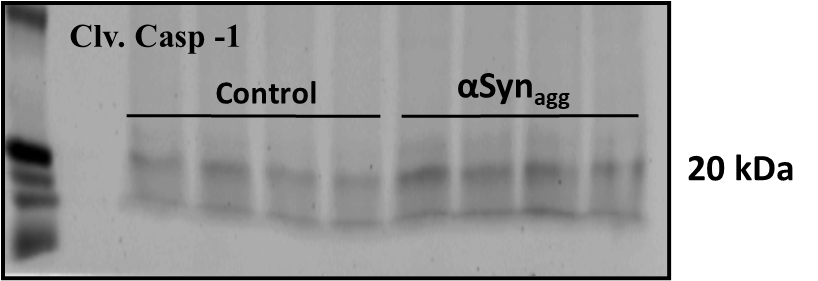

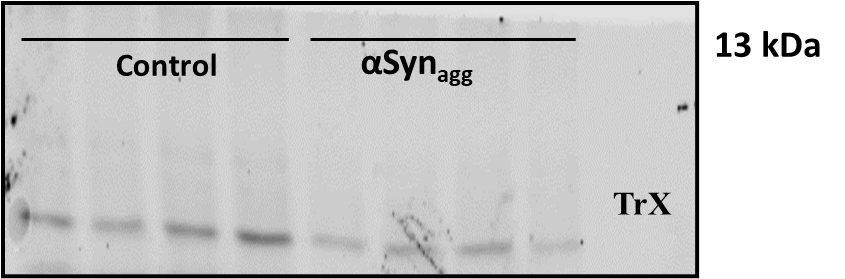
**

**
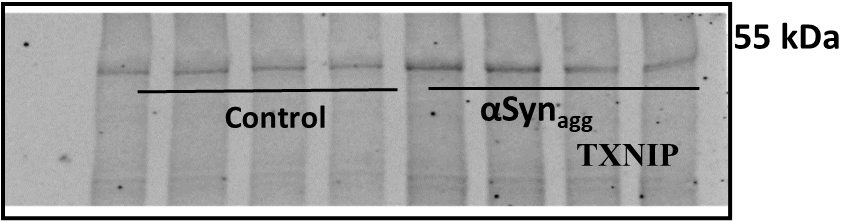

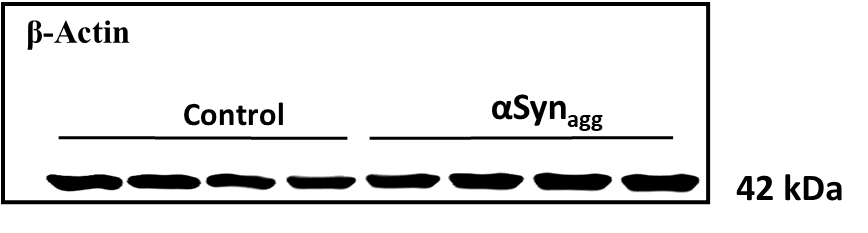
**

**
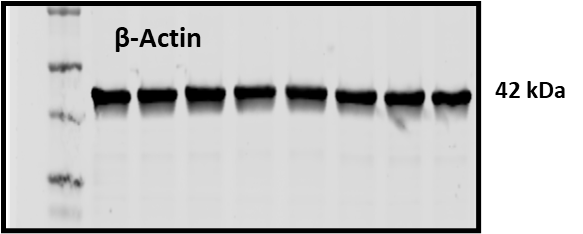
**

**Full blot images of Fig. 2**

**
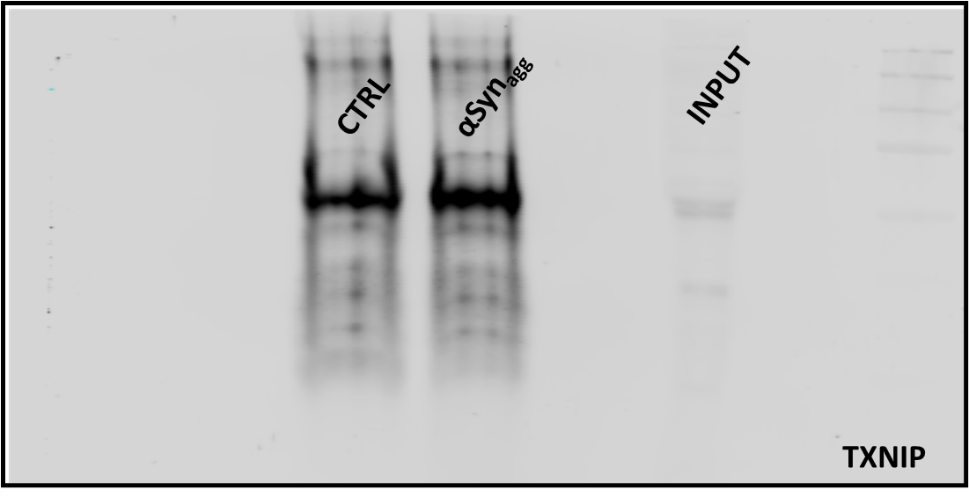
**

**Full blot images of Fig. 3**

**
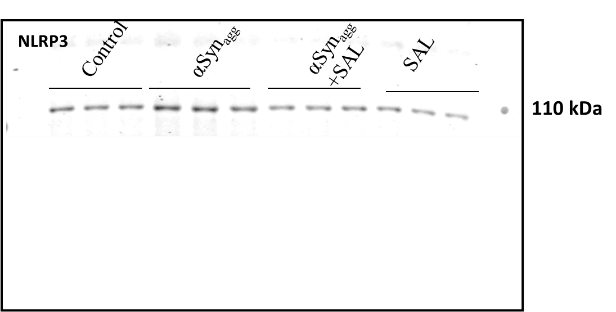

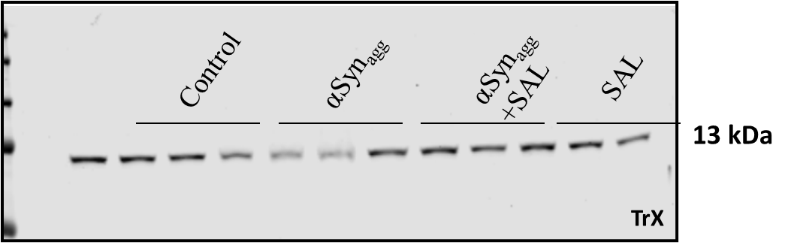
**

**
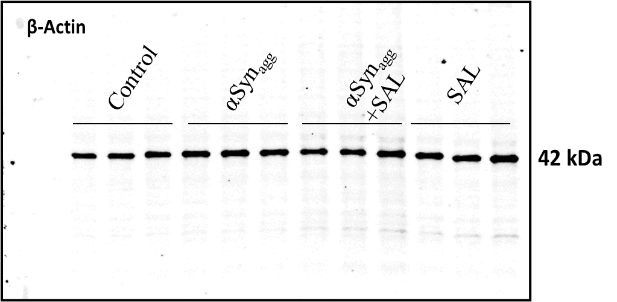

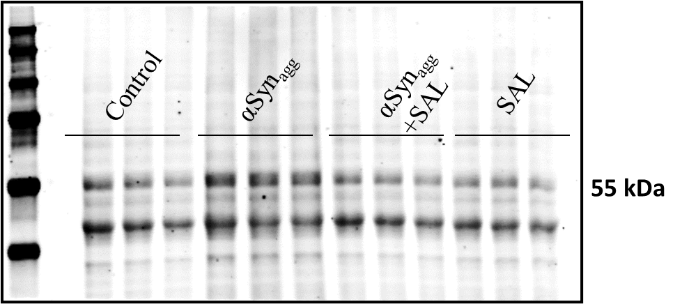
**

**Full blot images of Fig. 4**

**
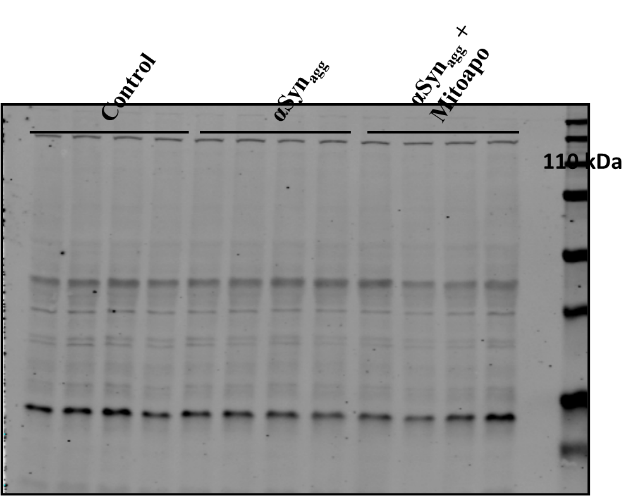

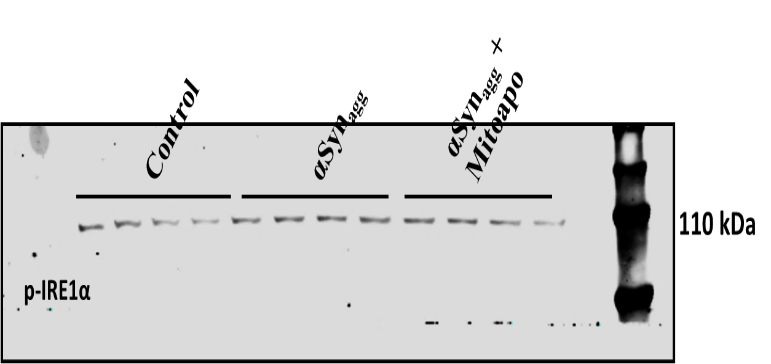
**

**
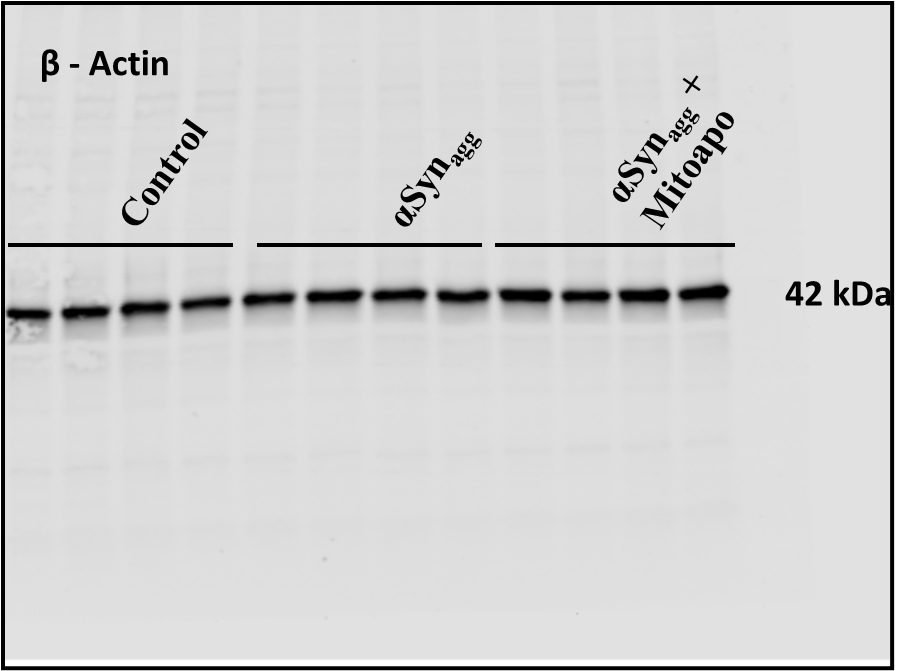

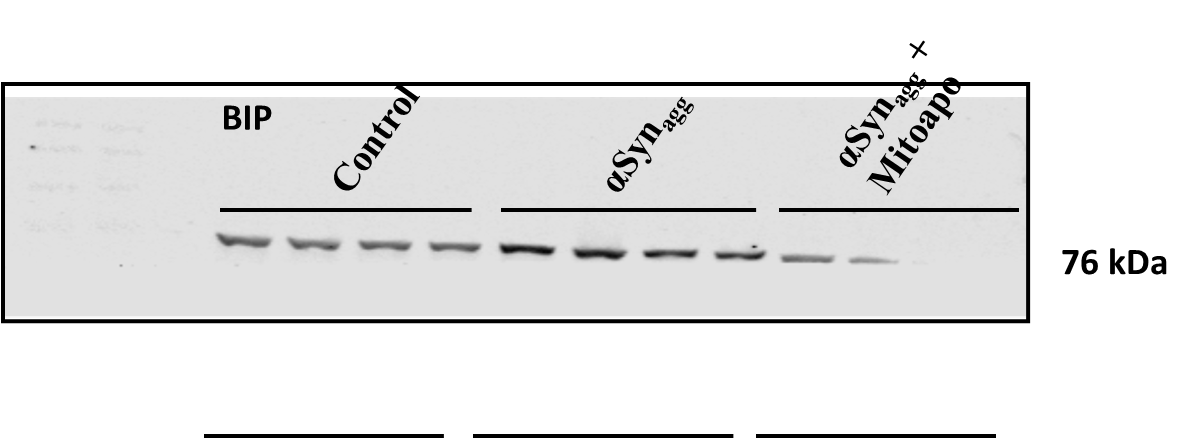
**

**
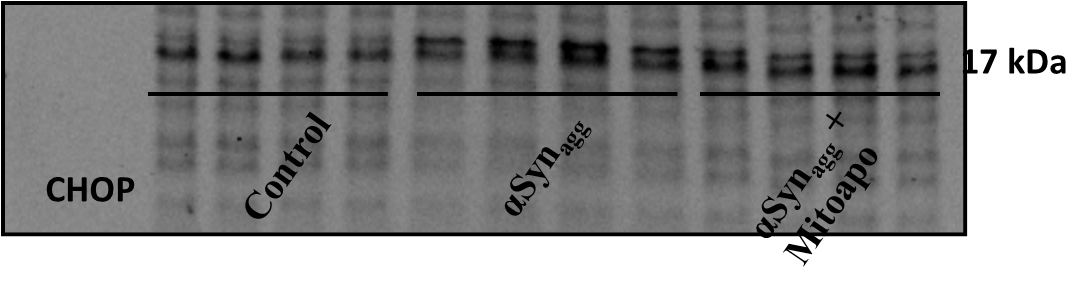

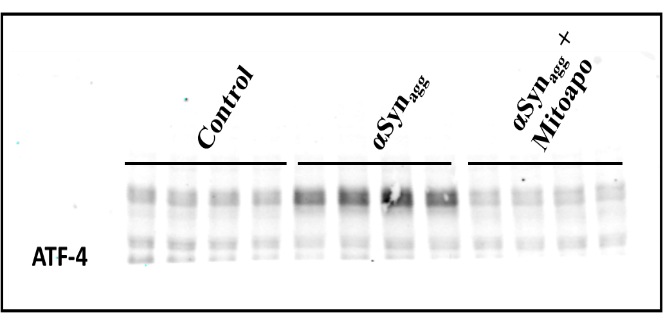
**

**
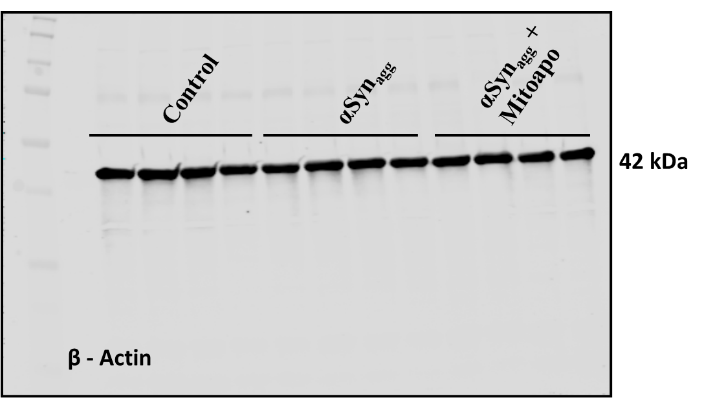
**

**Full blot images of Fig. 5**

**
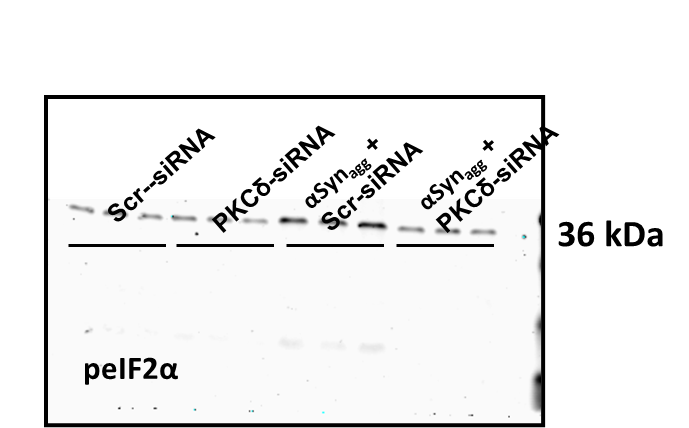
**

**
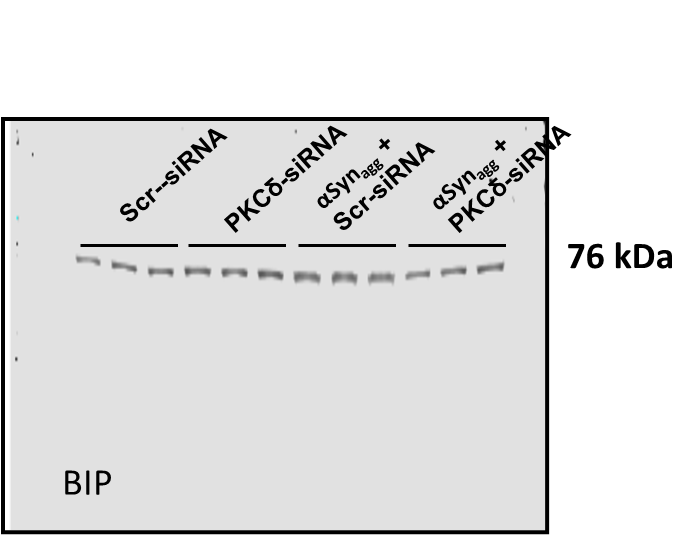
**

**Full blot images of Fig. 6**

**Full blot images of Fig. 7**

**(C)**

****  ****


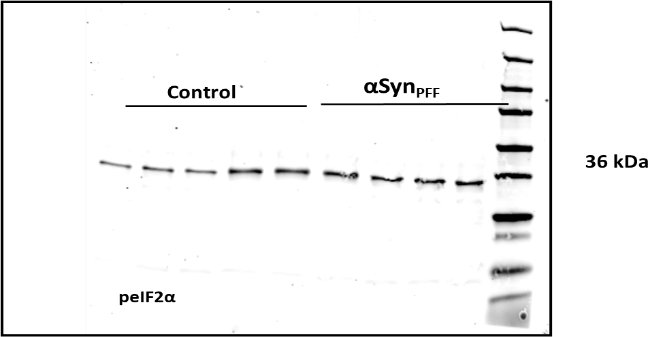


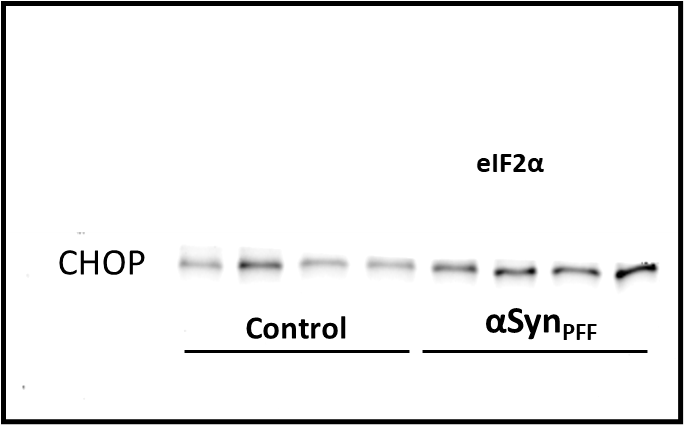


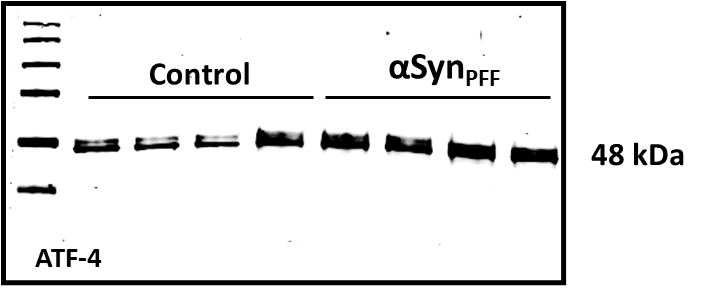

**Full blot images of Fig. 9**

**Supplementary Fig 4.** Full length Western blot images.

**Supplementary Table 1.** List of antibodies used for this study.

| **Antibody** | **Company** | **Catalog number** |
| --- | --- | --- |
| Ms pAb Caspase-1 | Adipogen | AG-20B-0042 |
| Rb mAb TXNIP | Cell signaling | 14715S |
| TH | Millipore | MAB318 |
| Rb pAb TrX | abcam | ab86255 |
| BIP | Cell Signaling | 3177S |
| CHOP | Cell Signaling | 5554S |
| αSyn | BD Biosciences | 610787 |
| Rb mAb p-PKCδ | Cell Signaling | 2058S |
| Rb mAb PKCδ | Cell Signaling | 2055S |
| Rb mAb ATF-4 | Cell Signaling | 11815S |
| Rb mAb ATF-6 | Cell Signaling | 65880S |
| Rb mAb p-IRE 1α | Cell Signaling | 3294S |
| Rb mAb p-eIF2α | Cell Signaling | 9721S |
| Ms mAb NLRP3 | Adipogen | AG-20B-0014-C100 |
| Rb pAb ASC | Adipogen | AG-25B-0006-C100 |

**Supplementary Table 2.** List of available qRT-PCR primer sequences used in this study.

| **Primer** | **Sequence** |
| --- | --- |
| TNF-α | Forward CCACCACGCTCTTCTGTCTAC  Reverse AGGGTCTGGGCCATAGAACT |
| IL-6 | Forward ACCAGAGGAAATTTCAATAGGC  Reverse TGATGCACTTGCAGAAAA |
